# Molecular Composition and Kinetics of B Cells During Ibrutinib Treatment in Patients with Chronic Lymphocytic Leukemia

**DOI:** 10.3390/ijms252312569

**Published:** 2024-11-22

**Authors:** Sólja Remisdóttir Veyhe, Oriane Cédile, Sara Kamuk Dahlmann, Jakub Krejcik, Niels Abildgaard, Thor Høyer, Michael Boe Møller, Mads Thomassen, Karen Juul-Jensen, Henrik Frederiksen, Karen Dybkær, Marcus Høy Hansen, Charlotte Guldborg Nyvold

**Affiliations:** 1 Haematology-Pathology Research Laboratory, Research Unit for Haematology and Research Unit for Pathology, University of Southern Denmark and Odense University Hospital, 5000 Odense, Denmark; solja.remisdottir.veyhe@rsyd.dk (S.R.V.); charlotte.guldborg.nyvold@rsyd.dk (C.G.N.); 2 Centre for Cellular Immunotherapy of Haematological Cancer Odense (CITCO), Odense University Hospital, 5000 Odense, Denmark; 3 Department of Haematology, Odense University Hospital, 5000 Odense, Denmark; 4 Odense Patient Data Explorative Network (OPEN), Odense University Hospital, 5000 Odense, Denmark; 5 Department of Hematology, Aalborg University Hospital, 9000 Aalborg, Denmark; 6 Department of Clinical Medicine, Aalborg University Hospital, 9000 Aalborg, Denmark; 7 Department of Pathology, Odense University Hospital, 5000 Odense, Denmark; 8 Department of Clinical Genetics, Odense University Hospital, 5000 Odense, Denmark; 9 Clinical Genome Center, Department of Clinical Research, University of Southern Denmark, 5000 Odense, Denmark

**Keywords:** chronic lymphocytic leukemia, ibrutinib, clonal stability, treatment efficacy, lymphocytosis, molecular profiling

## Abstract

Chronic lymphocytic leukemia (CLL) is characterized by the accumulation of B cells due to constitutive B-cell receptor (BCR) signaling, leading to apoptosis resistance and increased proliferation. This study evaluates the effects of the Bruton Tyrosine Kinase (BTK) inhibitor ibrutinib on the molecular composition, clonality, and kinetics of B cells during treatment in CLL patients. Employing a multi-omics approach of up to 3.2 years of follow-up, we analyzed data from 24 CLL patients, specifically focusing on nine patients treated with ibrutinib monotherapy. In this study, clonal stability was observed within the ibrutinib-treated group following an effective initial clinical response, where clonotype frequencies of residual CLL cells remained high and stable, ranging from 74.9% at 1.5 years to 87.7% at approximately 3 years. In contrast, patients treated with the B-cell lymphoma 2 (BCL2) inhibitor venetoclax exhibited substantial reductions in clonal frequencies, approaching molecular eradication. Deep whole-exome sequencing revealed minimal genomic progression in the ibrutinib group, maintaining somatic drivers and variant allele frequencies (VAF) above 0.2 throughout treatment. At the single-cell level, the NF-κB pathway inhibition and apoptotic signals were detected or even augmented during treatment in ibrutinib-treated patients. These findings may corroborate the role of ibrutinib in stabilizing the genomic landscape of CLL cells, preventing significant genomic evolution despite maintaining a high clonal burden within the residual B-cell compartment.

## 1. Introduction

Ibrutinib and other Bruton Tyrosine Kinase (BTK) inhibitors have transformed the treatment landscape for chronic lymphocytic leukemia (CLL) by targeting B-cell receptor (BCR) signaling, a central component in CLL cell survival and proliferation. In its method of action, ibrutinib irreversibly blocks BCR signaling, thus preventing CLL cell proliferation, migration, and adhesion [1,2,3,4]. One of the major pathways affected by continuous BCR signaling is NF-κB, which promotes cell survival and proliferation [5,6]. As chronic lymphocytic leukemia (CLL) is characterized by the accumulation of anergic lymphocytes in the blood, lymphatic system, and bone marrow, continuous oral BTK inhibitor administration brings the clonal expansion under manageable control. CLL is an often indolent malignancy, and for decades, immunochemotherapy has been the standard of care for many patients requiring treatment. However, targeted therapies, such as BTK and BCL2 inhibitors, are increasingly being used to manage both low- and high-risk CLL patients, such as those marked by del(17p) or mutated *TP53*.

The principal factors driving CLL pathogenesis, other than proliferation mediated through BCR signaling [7], are interaction with the microenvironment and resistance to apoptosis due to overexpression of B-cell lymphoma 2 (*BCL2*), primarily driven by the loss of regulatory microRNAs (miR-15a and miR-16-1) due to chromosome 13q14 deletion [8]. This BCL2 protein is the direct target of venetoclax [9]. Both therapies have shown efficacy and safety across first-line and relapse settings, as well as being applicable therapy for high-risk patients [10,11,12,13,14]. Moreover, as BTK inhibition is suggested to increase the dependency of the BCL2 survival protein [15], several studies have focused on combining the two [16,17,18,19], including the ongoing phase III CLL17 trial (EudraCT: 2019-003854-99).

In contrast to the pronounced clonal reduction in non-tyrosine kinase inhibitors, such as venetoclax, the ibrutinib administration is associated with a treatment-induced surge in peripheral lymphocytes [2,20], followed by an often prolonged lymphocytosis. At treatment initiation and within hours of administration, CLL cells are released from their protective microenvironments into the bloodstream [20,21]. Although this transient lymphocytic spike often resolves within weeks, the persistence of malignant clones over extended treatment periods is marked by an inability to achieve complete molecular eradication. The molecular composition and kinetics of the residual cells are not yet completely understood [2,21,22,23,24]. Complete remissions are more frequently achieved for patients receiving venetoclax mono- or combination therapy than for patients treated with ibrutinib alone [9,25,26,27]. On the contrary, venetoclax directly induces apoptosis by targeting the essential survival protein BCL2. Acting synergistically with CD20-targeting immunotherapy, venetoclax induces a complete and often durable response despite a time-limited duration of treatment [28]. Ibrutinib-induced downregulation of the CD20 antigen on CLL cells [29] may partially explain why the combination of ibrutinib with rituximab is not synergistic [25].

Previous studies have shown that ibrutinib can control CLL progression with minimal genomic evolution, stabilizing the genomic landscape even in high-risk patients [5,28]. However, the long-term molecular effects of ibrutinib on clonal composition and the potential for clonal evolution in a molecular framework remain to be fully explored. This relevance is emphasized by the study of Landau et al., showing that some change in the clonal fraction occurs in almost one-third of the patients [30]. Given the reasonable clinical response rates of both ibrutinib and venetoclax-based therapies, it is imperative to understand their differential impact on the CLL cellular compartment, profiled at the molecular level.

This study aims to provide new insights into how these treatments affect the clonal architecture of CLL by comparing the molecular kinetics of ibrutinib with venetoclax-treated patients. Understanding the endurance and longevity of malignant clones during ibrutinib treatment and identifying molecular signatures associated with clonal stability or progression may guide future therapeutic strategies in the long-term control of CLL. To address these gaps, we used a multi-omics approach to analyze the clonal stability, molecular composition, and kinetic profiles of CLL cells in patients treated with ibrutinib over a follow-up period of up to 3.2 years.

## 2. Results

The study included 24 patients with a median age of 59 years (range 43–79) at CLL diagnosis; both treatment-naive and previously treated patients were included. The cohort consisted of patients from two treatment groups: a BTK inhibitor treatment-focused group, including nine patients who received ibrutinib monotherapy, and a venetoclax control group, with 14 patients treated with a combination of rituximab and venetoclax and one patient receiving the combination of venetoclax and ibrutinib (Table 1 and Table S1). It was assessed that the slight difference in patients receiving earlier treatment (*p*_Fisher’s_ = 0.048) did not affect the aim of the study.

To investigate the molecular kinetics and stability of these patients diagnosed with CLL, the clonal composition of the lymphocytes was determined before treatment initiation and at subsequent follow-up time points within 0.5–3.2 years of treatment. At study termination, six patients were still receiving ibrutinib treatment. Among the patients discontinuing ibrutinib, the treatment duration was 189, 1166, and 1211 days. Four patients receiving venetoclax were still receiving treatment at the time of study termination. The treatment duration for patients completing or discontinuing therapy was 181–1460 days (Figure S1).

### 2.1. Lymphocyte Kinetics During Treatment

**Figure 1:**
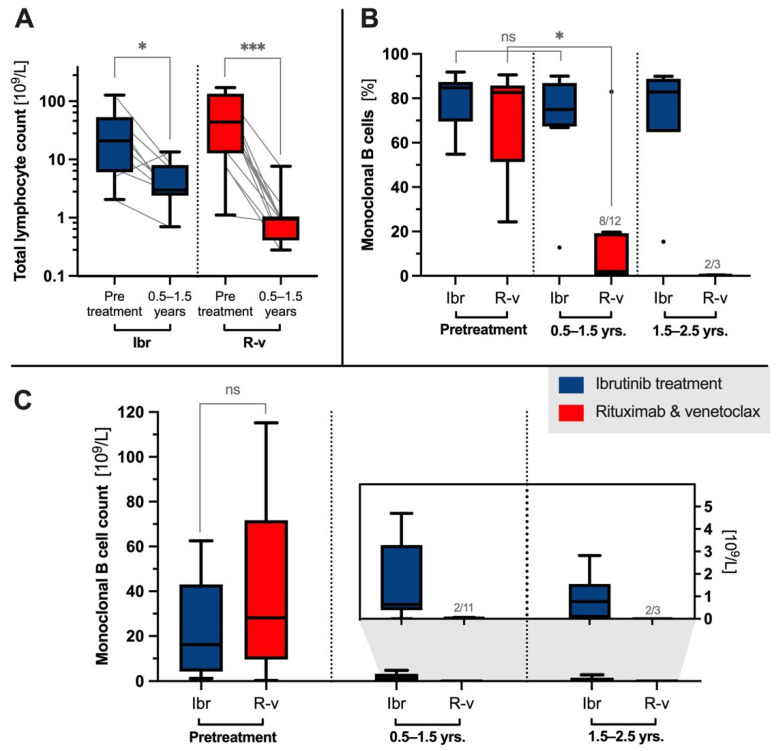
Clonal kinetics following administration of ibrutinib (Ibr) or rituximab–venetoclax (R–v). Total lymphocyte counts before treatment in ibrutinib-treated versus R–v (*p*_ranksum_ = 0.21), with significant (signed-rank) median reductions in lymphocyte counts from 20.90 × 10⁹/L (n = 9) to 2.98 × 10⁹/L (n = 9) for ibrutinib and from 43.99 × 10⁹/L (n = 14) to 0.95 × 10⁹/L (n = 12) for rituximab–venetoclax (R–v) at follow-up of 0.5 to 1.5 years (**A**). The monoclonal burden was 75% at first follow-up in the ibrutinib group (n = 9) compared with 1.99% in the R–v group (n = 12), with residual disease undetectable in 4 of 12 patients in the R–v group at 0.5 to 1.5 years (**B**). Estimated monoclonal CLL cell counts from flow cytometry: 16.2 × 10⁹/L (n = 9) for ibrutinib and 28.2 × 10⁹/L (n = 15) for R–v before treatment, decreasing to 0.6 × 10⁹/L for ibrutinib (n = 9) and near the detection limit for R–v (n = 11) during follow-up of 0.5 to 1.5 years (**C**). Abbreviations *: *p* < 0.05, ***: *p* < 0.005, ns: not significant. Dotted lines denote separate treatment groups or follow-up intervals.

Before treatment initiation, the median lymphocyte count was 37.1 × 10^9^/L (25% percentile: 7.97 × 10^9^/L, 75% percentile: 68.00 × 10^9^/L) with no significant difference between the treatment groups (*p*_ranksum_= 0.21). The therapeutic intervention decreased the lymphocyte count at the first follow-up (0.5–1.5 years) to 2.98 × 10^9^/L for the ibrutinib cohort (*p*_signed-rank_ = 0.019, Figure 1A) and to 0.95 × 10^9^/L for the R–v cohort (*P*_signed-rank_ < 0.001). At this time point, flow cytometric analysis showed that CD19+ B cells constituted 31.3% of the total lymphocytes in ibrutinib-treated patients (Table S5A), in contrast to being non-detectable in the majority of the R–v control cohort (Table S5B). Ibrutinib patients remained steady from 1.5–3.2 years, with CD19+ monoclonal B cells constituting 24.2% of the lymphocyte population (Table S5A).

### 2.2. Clonotype Stability

Investigating the clonal composition of residual B cells during treatment, the LymphoTrack analysis specifically measured clonal cells within the total pool of cells with rearranged *IGH* genes. The nine patients treated with ibrutinib (28 samples) showed stability of the clonal burden compared with the 15 patients receiving venetoclax (35 samples). Initially, the percentages of clonotypic *IGH* rearrangements were similar before treatment with ibrutinib and venetoclax across both treatment groups, at 84.7% (range: 54.8%–91.8%) for ibrutinib and 82.6% (24.4%–90.6%) for venetoclax (Figure 1B). The *IGHV* repertoire is shown in Supplementary Table S3A,B. A notable response difference between the two groups was observed during follow-up (Figure 1B), with ibrutinib-treated patients showing unaltered median clonotype frequencies of 75.0% (0.5–1.5 years, *p*_signed-rank_ = 0.328), 82.9% (1.5–2.5 years), and 87.7% (2.5–3.2 years), indicating robust clonotype stability. In contrast, the R–v-treated group exhibited polyclonality in the low number of B cells present (0.5–1.5 years: 1.99%, *p*_signed-rank_ = 0.016, 1.5–2.5 years: 0.24%) with no measurable residual disease in 5 out of 15 patients.

From the total lymphocyte count multiplied by the CD19+ lymphocyte fraction and the clonotype burden from the targeted sequencing of *IGH* rearrangements, an estimate of the CLL cell counts prior to ibrutinib or R–v treatment was calculated to be 26.6 × 10^9^/L (Figure 1C, 25% percentile: 6.5 × 10^9^/L, 75% percentile: 44.5 × 10^9^/L), dropping to 0.6 × 10^9^/L for ibrutinib and close to the detection limit for R–v (Figure 1C, insert). Notably, in one R–v patient, the diagnostic clone constituted the majority of the residual B cells (83.0%).

The detection sensitivity using clonotyping was estimated at 10^−4^, with a median confidence level of 95.2% based on 0.1–5.6 µg DNA input and a median of 6 × 10^5^ reads. The confidence levels were estimated using an empirically validated Poisson method for clonotype detection [31].

### 2.3. Clonal Evolution at the Genomic Level

To investigate this clonal stability at the genome level, high-resolution whole-exome sequencing (WES) of the B-cell compartments was performed for 38 samples, each paired with a corresponding T-cell control subset. Ten out of 11 baseline/pretreatment samples were derived from PBMCs, while nucleic acids were extracted from enriched B cells in all follow-up samples. The samples originated from seven patients undergoing ibrutinib treatment and four patients treated with venetoclax. Using flow cytometry, the purity of CD19+ enriched cells was estimated to be 87.0% (77.9–94.1%) for the samples, while the purity for the T-cell control paired samples was 72.6% (31.1–73.7%), with a maximum B-cell contamination of 0.02%.

**Figure 2:**
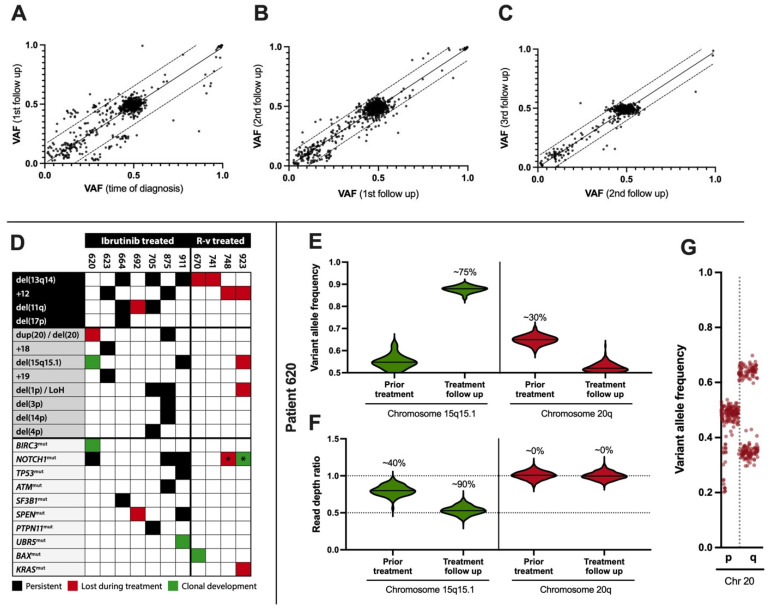
Genomic stability following ibrutinib treatment. Correlation between somatic variants in CD19+ cells prior to treatment and at first follow-up ((**A**), ρ_Pearson_ = 0.86, slope 0.98, *p*_t-statistics_ < 10^−4^, mean follow-up 11 months, n = 7), between first and second follow-up ((**B**), ρ = 0.95, slope 0.99, *p* < 10^−4^, 20 months, n = 7), and between second and third follow-up ((**C**), ρ = 0.94, slope 0.98, *p* < 10^−4^, 34 months, n = 5). Cytogenetic markers del(13q14) and trisomy 12 following rituximab–venetoclax (R–v) treatment and copy-number alterations (CNAs) for ibrutinib-treated patients (**D**). *NOTCH1* frameshift mutation c.7541–7542del (p.P2514Rfs*4, COSV53024776, GRCh38, 9:136496197 CAG>C) was detected with a variant allele frequency of 0.21 in R–v patient 923 at first follow-up (marked *). The same mutation was detected with a variant allele frequency decreasing to 0.03 in R–v patient 748 (*). Allelic imbalances and CNAs on chromosome 15q and 20q, including the *BIRC3* mutation, were found for patient 620. The clonal burden of chromosome 15q deletion increased from less than 50% to more than 50% during treatment (**E**,**F**) and copy-neutral loss of heterozygosity at 20q during follow-up (solid lines separate chromosome 15q and 20q, dotted lines denote the theoretical ratios for disomy, 1.0, and deletion, 0.5). Allelic imbalance on chromosome 20 was evaluated at 30% burden ((**G**), a dotted line separates chromosomal p and q arm).

The median number of putative somatic variants found at least once after treatment over the longitudinal timeframe was 35 in the ibrutinib group (variant allele frequencies (VAF) > 0.1, coverage of 794) and 12 for the venetoclax group, supporting an eradication of malignant B cells in the venetoclax group. Notably, somatic variants were largely absent at high frequencies (VAF > 0.2), in contrast to the 15 observed in the ibrutinib group. Furthermore, only 25% of variants in the venetoclax group were observed more than once, compared with 82% for ibrutinib. This revealed a significant correlation between putative somatic VAF at diagnosis and during follow-up for ibrutinib-treated patients (ρ_Pearson_ = 0.83, *p*_t-statistics_ < 10^−4^, Figure 2A–C). Several somatic mutations in genes were central to the involvement of CLL or in B-cell signaling pathways, e.g., *ATM* (11:108251037 G>A and 11:108365085 C>G), *BIRC3* (11:102331211 A>AG and 11:102336973 CAAAG>C), *NOTCH1* (9:136496196 CAG>C), *PTPN11* (12:112486553 G>A), *SF3B1* (2:197402767 C>G), *SPEN* (1:15930057 GAGGATCC>G), *TP53* (17:7675088 C>T), and *UBR5* (8:102296933 TACA>T), and were confirmed at the transcriptional level. The median number of SNP-filtered somatic variants per sample was 60 with a median VAF of 0.46 (see Data Availability Statement).

At the structural level, WES confirmed clinical cytogenetic analyses of 11q and 13q14 deletions, trisomy 12, and 17p (*TP53*) status. The analyses were based on changes in read depth ratios and allelic imbalances, with only minor clonal progression (Figure 2D). However, for the ibrutinib-treated patients, some differences were noticed; one (pt. 620) showed a complete clearance of duplicated 20q resolved by allelic imbalance, superseded by a 2 Mb deletion of 15q15.1 (Figure 2D–G). At the second follow-up, another patient (pt. 692) showed clearance of the 11q deletion, affecting the *ATM* tumor suppressor gene (Figure 2D). Finally, the UBR5 mutation appeared at the first follow-up (pt. 911). Apart from these minor changes, no prominent features of clonal progression during treatment intervention were found, and the existing copy-number alterations were marked by resilience to treatment and a high clonal burden. In contrast, although the R–v group also displayed clinically confirmed chromosomal copy-number alterations, e.g., del(13q14) and trisomy 12, no chromosomal alterations sustained treatment, except for a *NOTCH1* mutation gained during treatment (pt. 923, Figure 2D).

### 2.4. Single-Cell Resolution of the CLL Compartments During Ibrutinib

**Figure 3:**
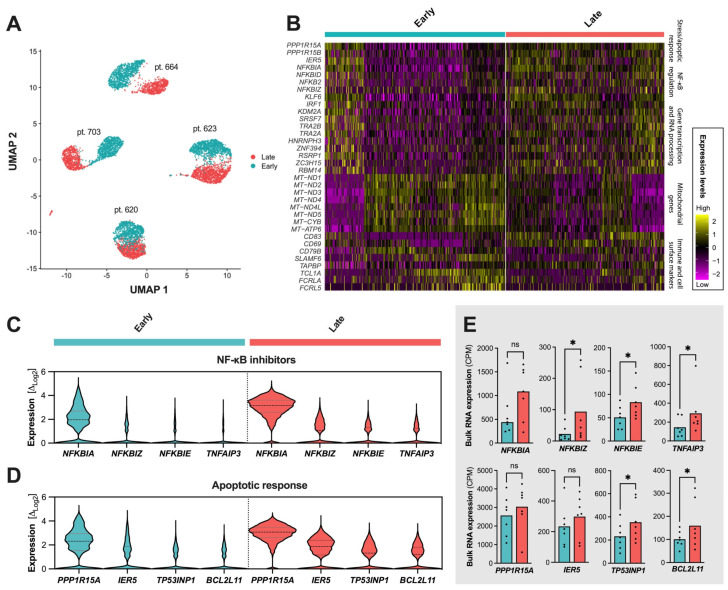
Transcriptional profile of ibrutinib treatment responses. Transcriptional landscape of single cells at early therapeutic follow-up (cyan: mean follow-up of 14 months) and prolonged administration (pale red: 36 months) (**A**–**E**). Significant expressional changes were identified in genes relating to the NF-κB inhibitor family, stress and apoptotic response, or mitochondrial oxidative phosphorylation (**B**). Increased expression or number of cells expressing *NFKBIA* and other NF-κB inhibitors after prolonged ibrutinib administration (**C**). Increased expression of *PPP1R15*, *IER5*, *TP53INP1*, and *BCL2L11*—genes involved in apoptotic regulation—at late ibrutinib response (**D**). Bulk RNA sequencing confirming upregulation trends in late follow-up samples for markers involved in B-cell signaling and survival (**E**). Abbreviations *: *p* < 0.05, ns: not significant. Dotted lines in (**C**) separate early and late response profiles. Dots in (**E**) show individual expression levels for the bulk RNA sequencing samples when expressed.

To further explore the apparent clonal stability of the CLL cells during follow-up, we investigated four of the ibrutinib-treated patients from whom samples were available at three follow-up time points using single-cell RNA expression, amounting to a total of 165,148 analyzed cells. These cellular transcriptome subsets were divided into early (~1 year), intermediate (~2 years), and late responses (~3 years). The most pronounced significant differences were found between early and late treatment follow-up, foremost involving stress or apoptotic response, pathway modulation, transcription factors, mitochondrial genes, or surface and immune markers (Figure 3A,B).

While the expression profiles showed no evident subclonal populations or clonal evolution at the single-cell level, several NF-κB inhibitor gene family members or other inhibitors were augmented at the late ibrutinib treatment follow-up, with *NFKBIA*, *NFKBIE*, *NFKBIZ*, and *TNFAIP3* being the most prominent members (Figure 3C, *NFKBIA*: Log_2_ fold change (Δ) = 2.15, *p* ≈ 0; *NFKBIE*: Δ = 1.90, *p*_ranksum_ < 10^−45^; *NFKBIZ*: Δ = 1.72, *p* < 10^−52^; *TNFAIP3*: Δ = 1.84, *p* < 10^−43^; all Bonferroni-corrected). Interestingly, 17% of the most significantly altered in single-cell RNA expression (17/100) directly overlapped with *genes regulated by NF-kB in response to TNF* using GSEA [32,33] and the Molecular Signatures Database (Human MSigDB v2024.1.Hs, UC San Diego and Broad Institute, *P*_hypergeometric_ < 6.24 × 10^−22^, false discovery rate (FDR) = 4.78 × 10^−18^).

Exploring the significantly altered genes further, enrichment was associated with transcriptional changes in the *apoptotic process* or *apoptotic signaling pathway* (gene set M45068 and M12660), *programmed cell death* (M46792), *response to stress* (M42745), etc. (Table 2). Of note, these profiles were not attributed to cellular decay from laboratory handling, with an estimated 95% viable cells before library generation and low single-cell mitochondrial transcriptional counts of 1.5% in the early follow-up and 0.9% in the late follow-up.

As the most noticeable markers of cellular stress response, the genes involved in apoptosis or cell cycle regulation were increased in the single-cell expression of *PPP1R15A*, *IER5*, *TP53INP1*, and *BCL2L11* (Figure 3D). Bulk RNA sequencing quantitatively confirmed an increased expression of the NF-κB inhibitor members, with a mean fold change of 2.38 (range 1.63–4.15) and aforementioned regulatory markers (Figure 3E).

Other transcriptional markers included genes coding for key transcription factors (e.g., *IRF1* and *KLF6*), mitochondrial oxidative phosphorylation, cell surface markers (e.g., *CD83*, *CD69*, and *CD79B*), markers of cellular organization, migration, and cell adhesion (1.75 < Δ < 3.42; 10^−111^ < *p*_ranksum_ < 10^−41^; Bonferroni-corrected). However, these markers warrant further analyses or cohort expansion.

## 3. Discussion

This study aimed to investigate the molecular composition of CLL cells in patients receiving ibrutinib treatment. Previous studies have shown that overall response rates are generally high (43–96%) across age groups, in previously untreated or relapsed/refractory patients, and across risk status [2,10,34,35,36]. Our ibrutinib-treated cohort demonstrated a profound initial reduction in the circulating lymphocyte concentration; for the venetoclax-treated patients, this reduction was even more pronounced. Despite the high response rates associated with ibrutinib monotherapy, we show that the malignancy persists, in agreement with previous reports 13−17, and that the residual cells are largely monoclonal with pronounced stability.

As such, flow cytometric analyses and the assessment of the genomically rearranged immunoglobulin heavy chain loci confirmed a sustained high frequency of monoclonal peripheral B cells in patients half a year post ibrutinib initiation compared with those receiving rituximab and venetoclax. In the latter group, the clonal populations were markedly reduced or below the limit of detection. Estimates indicated the presence of several hundred million to a few billion monoclonal CLL cells per liter, persisting to three years of follow-up while receiving BTK inhibitor monotherapy. These specific cells demonstrated kinetic stability and molecular perseverance, as evidenced by consistent somatic variant detection from pretreatment to follow-up, except in one patient who fell below the detection threshold of our deep sequencing of the coding genome at a 1.5-year treatment duration. Here, the CD19+ cells constituted less than 1% of the total lymphocyte population, comprising a mere 15% monoclonality.

Transient lymphocytosis is a well-documented effect of BTK inhibitors, where CLL cells and non-malignant B cells are released from the bone marrow niche and into the bloodstream. In a study on kinetic profiling of lymphocytosis, Barrientos et al. showed that lymphocytosis resolved in 94–95% of the patients with a median duration of 12 to 14 weeks [23]. Lymphocytosis was reported as exceeding a 50% increase in lymphocyte count compared with the baseline or absolute cell count above 5 × 10^9^/L. These findings also corroborated the early results by Byrd et al., where the initial lymphocytosis decreased after four weeks of ibrutinib treatment and gradually normalized [2]. Our findings show that while clinical remission is achievable and sustainable, monoclonal lymphocytosis at the molecular level persists, with about 80% of clonally rearranged *IGH*-positive peripheral B cells and concordant with the somatic variant burden being sustained after at least 1.5 to 2.5 years of continuous treatment. Conversely, venetoclax-treated patients showed deep responses, with some even achieving complete molecular responses with no detectable residual clonal B cells.

Ibrutinib has significantly enhanced overall survival rates, particularly as a first-line treatment, compared with other therapies [10,37], although incapable of cellular eradication. The decrease in CLL cell counts after ibrutinib therapy is accompanied by stability and minimal genomic progression, which aligns with findings by Byrd et al. [24] and others. Several reports support the effectiveness of disease suppression over extended periods, potentially prolonged even after cessation. Patients discontinuing ibrutinib have demonstrated a median progression-free survival (PFS) of 24–25 months, observed in both the A041202 [37] and E1912 [38] trials.

It is suggested that ibrutinib is at least capable of regressing certain CLL genotypic features, as seen by the seeming subclonal eradications. In one of the patients we investigated, this included clearance of del(11q) and loss of the mutation affecting the negative regulator of the Notch pathway member *SPEN*. In contrast, only minor clonal progression is directly demonstrated, such as the de novo mutated *BIRC3* and del(15q15.1) observed for another patient. Currently, the closest estimate is that a clonal shift from ibrutinib treatment, reported by Landau et al., may be expected in approximately 31% of the patients in the first year of treatment [30].

Albeit a limited cohort, all ibrutinib-treated patients demonstrated a remarkably stable genome, with the vast majority of genomic aberrations also present at follow-up. Previous studies have demonstrated a substantial number of somatic mutations in CLL, which may be in the range of thousands [39]. In the current study, fewer than a hundred individual somatic variants were exclusively found in CD19+ cells, with a third of these DNA variants at an allele frequency of 0.2 or more being propagated at the transcriptional level and consistently detected between follow-up time points.

It may be suggested that the modulation or even inhibition of the NF-κB signaling pathway here is a direct result of ibrutinib treatment [30,40,41]. This observation is in line with the findings by Rendeiro et al. that showed a reduction of chromatin accessibility at the NF-κB binding site and a downregulation of the NF-κB signaling pathway at the single-cell level [42]. In our study, this is particularly evident from the increased expression of the NF-κB inhibitor *NFKBIA*. Perhaps supporting this notion, we observe an upregulation and enrichment of proapoptotic response genes and negative cell cycle regulators, including *PPP1R15*, *IER5*, *TP53INP1*, and *BCL2L11*. Interestingly, Bonfiglio et al. [43] recently revealed NF-κB signaling pathway gene mutations in patients who relapsed while undergoing ibrutinib treatment. This addresses the substantial number of patients without resistance mutations in the *BTK* and *PLCG2* genes, which were also absent in our study. Subsequent functional studies have found that diminished expression of *NFKBIE* undermines the effectiveness of ibrutinib, mitigating its known benefits, such as proliferation inhibition, apoptosis induction, and increased cellular mobilization, both in laboratory and clinical settings [44]. Collectively, this supports the relevance and importance of the BCR and downstream NF-κB-mediated signaling during ibrutinib treatment. However, further studies are needed to shed light on the kinetics and durability of the potential negative NF-κB regulation.

In conclusion, while ibrutinib significantly reduces CLL cell counts with clinical remission, the perseverance of malignant clones underscores that eradication is generally not achievable using this treatment. Hence, our study further highlights the need for research to unravel the kinetics of enduring malignant B cells and their shifts in central pathway signaling in ibrutinib-treated patients in order to enhance treatment efficacy and outcomes.

## 4. Materials and Methods

### 4.1. Patient Cohort and Sample Collection

CLL patients were included over a period of 2.5 years before treatment with ibrutinib or venetoclax. Patient inclusion is outlined in Table S1, and longitudinal sample collection for clonotyping is illustrated in Figure S1. Treatment was assigned by the physicians based on guidelines and patient characteristics. Study eligibility required informed patient consent and sufficient sample material, resulting in a cohort of 24 patients (21 from Odense University Hospital and 3 from Aalborg University Hospital). Of these, 9 were treated with ibrutinib, while 15 received a combination of rituximab–venetoclax (R–v) or, in one case, ibrutinib–venetoclax. The latter patient was categorized within the venetoclax-treated cohort. Four patients treated with venetoclax had previously been on ibrutinib therapy. None in the ibrutinib cohort had previously been treated with venetoclax.

Pretreatment samples were collected before treatment commenced (n = 11) or before administering ibrutinib or venetoclax in relapsed/refractory patients (n = 13). All subsequent samples were obtained during the treatment period, except in three instances where the samples were collected after the patients had discontinued R–v treatment at 0.5–1.5 years and 1.5–2.5 years, respectively. At these time points, no measurable residual disease was found. The study was conducted in compliance with the Declaration of Helsinki. Ethical approval was secured from the Regional Ethics Committee for the Region of Southern Denmark (approval number S-20160069).

### 4.2. Sample Processing and Storage

Blood samples collected in EDTA tubes during the clinical follow-up were centrifuged on a Ficoll gradient medium to isolate peripheral blood mononuclear cells (PBMCs). Cell lysates were prepared using RLT plus buffer (Qiagen, Hilden, Germany) or mRNA lysis buffer (Roche, Basel, Switzerland) and stored at −80 °C. Additionally, viable cells for sequencing analysis were cryopreserved in dimethyl sulfoxide and liquid N_2_. DNA and RNA extractions from lysed cells were performed using the AllPrep DNA/RNA mini or micro kits (Qiagen), with DNA concentrations determined by the Qubit 4.0 Fluorometer (Thermo Fisher Scientific, Waltham, MA, USA) and RNA integrity and concentration determined by the Bioanalyzer 2100 (Agilent Technologies, Santa Clara, CA, USA, Supplementary Methods).

### 4.3. Flow Cytometry on Blood

Flow cytometry staining, using the LST Euroflow panel [45,46] (Supplementary Methods, Table S2), and a designed panel (Table S3) were used to phenotypically characterize B, T, and NK cell populations and to assess the purity of the enriched B and T cells from the collected blood samples. Cells were acquired using the BD FACSLyric and BD FACSCanto instruments (BD Biosciences, Franklin Lakes, NJ, USA).

### 4.4. Cell Isolation and Purity Assessment

For purification and cell isolation, thawed PBMCs were enriched using the respective Pan B or Pan T Cell Isolation Kit (Miltenyi Biotec, Bergisch-Gladbach, Germany). Cell viability and counts were assessed with trypan blue or the NucleoCounter NC-202 (ChemoMetec, Allerød, Denmark). Enriched cells were either processed for single-cell RNA sequencing or lysed for further RNA/DNA extraction, with purity assessments conducted before and after isolation by flow cytometry.

### 4.5. IGH Sequencing

The LymphoTrack *IGH* FR1 assay for either S5/PGM or MiSeq (Invivoscribe, San Diego, CA, USA) was used for the clonotyping of 63 pretreatment and follow-up samples (Table S4A,B, Figure S1). IGH sequences from genomic DNA (input of 0.1–5.6 µg) extracted from PBMCs were first amplified and then sequenced by Ion GeneStudio S5 Prime systems (Thermo Fisher Scientific) or MiSeq (Illumina, San Diego, CA, USA) using an Ion 530 chip for S5/PGM and a V3 flow cell for MiSeq. Analysis was performed with the LymphoTrack Dx (v2.4.3–2.4.8) and MRD (v2.0.2, Invivoscribe) to evaluate the frequency of clonal cells to the total number of rearranged B cells. All clonotype sequences were confirmed using NCBI IgBLAST [47] A rough estimate of CLL cell counts was based on the total lymphocyte count as obtained by the Sysmex XN-series or XR-series (Sysmex Corporation, Kobe, Hyogo, Japan), the CD19+ fraction as obtained by flow cytometry (Figure S2, gating strategy), and the *IGH* clonotype burden (Table S4A,B).

### 4.6. Whole-Exome (WES) and RNA Sequencing

WES was performed for 11 patients, consisting of the diagnostic baseline sample and paired 1–4 follow-up time points per patient (38 samples in total), all paired with T-cell subsets as pseudo-germline control. Ten baseline samples were sequenced as PBMCs (CLL infiltration 80%, range 36–94%), as viable cells were unavailable. DNA and RNA were extracted from purified B or T cells in all other samples. Nucleic acid concentration and RNA integrity were measured for all samples as outlined. Whole-exome preparation was performed using the Twist Exome 2.0 hybridization and target enrichment kit (Twist Bioscience, South San Francisco, CA, USA), covering 36.5 Mb of protein-coding regions, and full transcript sequencing libraries were generated using Illumina Stranded mRNA Prep. The libraries were paired-end sequenced on the NovaSeq 6000 System (Illumina, San Diego, CA, USA). Whole-exome sequencing data were mapped with BWA [48], and RNA sequence mapping was performed using STAR2 [49]. Variant calling was performed in both DNA and RNA with GATK 4.3 [50]. All variant allele frequencies were derived from HaplotypeCaller (to assess allelic imbalance) or Mutect2 output (somatic variants). Downstream somatic variant annotation and filtration were performed using SnpEff (GRCh38.p13, dbNSFP v4.1a) and SNPsift (dbNSFP v4.1a) [51] with external data sources gnomAD (v3.1.2), ClinVar (11 December 2022) [52], and COSMIC [53] (v.98).

### 4.7. Single-Cell Transcriptomics

Between 1.84 × 10^6^ and 10.7 × 10^6^ enriched B cells from four ibrutinib-treated patients were fixed with the Chromium Next GEM Single-Cell Fixed RNA Sample kit (10x Genomics, Pleasanton, CA, USA). Libraries were generated using the Chromium Next GEM Single-Cell Fixed RNA Hybridization and Library kit (PN-1000475, 10x Genomics) and sequenced on NovaSeq 6000 (Illumina) using the S2 flow cell. Data analysis utilized Cell Ranger 7.2 (10x), R 4.3.1, and Seurat 5.

### 4.8. Statistics and Computations

Statistical analysis was performed using R 4.3.1 and Wolfram Language (Mathematica 13.3, Wolfram Research, Champaign, IL, USA). GraphPad Prism (v.10.1.2, GraphPad Software, Boston, MA, USA) was used for plotting and complementary statistical evaluation. Processing and analysis of sequencing data were conducted on the UCloud interactive high-performance computing system hosted by the eScience Center at the University of Southern Denmark.

## Figures and Tables

**Table 1:** Patient characteristics at diagnosis. The table presents diagnostic characteristics for patients undergoing treatment with ibrutinib (n = 9) and venetoclax (n = 15). Cytogenetic analyses were performed for 7 bone marrow samples, 1 blood sample, and 1 lymph node sample for the ibrutinib-treated group and 5 bone marrow samples, 6 blood samples, and 1 lymph node sample for the venetoclax cohort. Some of the patient characteristics were not available for all patients; therefore, the numbers do not always respectively add up to 9 and 15 for the two patient cohorts.

	Ibrutinib, n = 9	Venetoclax, n = 15	Statistical Difference
		(14 Rituximab–Venetoclax, 1 Ibrutinib–Venetoclax)	Fisher’s Exact (Categorical) and Rank-Sum (Continuous)
Sex M:F	7:2	9:6	*p* > 0.05
Age at diagnosis	59 (43–79)	59 (47–74)	*p* > 0.05
Binet stage A:B:C at diagnosis	4:3:2	8:4:0	*p* > 0.05
Binet stage A:B:C at treatment initiation	0:3:6	1:6:8	*p* > 0.05
Number of prior CLL treatments (0:1:2–3)	4:2:3	1:10:4	*p* = 0.048
Reported weight loss, fever, or night sweats	2/9	3/13	*p* > 0.05
**Cytogenetics**			
Del(17p)	0/9	0/13	*p* > 0.05
Del(11q)	3/9	4/13	*p* > 0.05
Trisomy 12	1/9	5/13	*p* > 0.05
Del(13q14)	6/9	5/13	*p* > 0.05
IGHV status (M-CLL:U-CLL) *	1:6	7:7	*p* > 0.05
TP53 mutation	1/4	0/6	*p* > 0.05
**Blood sample values**			
Hemoglobin (mmol/L)	7.7 (6.1–9.5)	8.7 (7.5–10.1)	*p* > 0.05
Leukocytes (WBC) (109/L)	48.6 (10.1–193)	18.4 (4–61.4)	*p* > 0.05
Thrombocytes/Platelets (109/L)	196.3 (55–372)	240 (116–433)	*p* > 0.05
Neutrophils (109/L)	3.6 (1.1–5.6)	3.3 (0.4–8.7)	*p* > 0.05
Lymphocytes (109/L)	32.3 (5.2–127)	7.5 (0.38–39.1)	*p* > 0.05
Beta-2-microglobulin (mg/L)	64.4 (3.1–227)	144 (2.6–344)	*p* > 0.05
Creatinine (µmol/L)	81.9 (72–106)	84 (47–105)	*p* > 0.05
Lactate dehydrogenase (LDH) U/L	232.9 (160–382)	193 (139–388)	*p* > 0.05
C-reactive protein (CRP) (mg/L)	3.3 (0.6–10)	1.7 (0.6–5)	*p*> 0.05

* M-CLL: mutated CLL and U-CLL: unmutated CLL.

**Table 2:** Gene set enrichment analyses based on the most significant single-cell expressional shifts (n = 100) from early (~1 year) to late response (~3 years) following ibrutinib treatment.

Gene Set Name Number of [Genes]	Genes in Overlap	*p*-Value	FDR
Hallmark TNFA signaling via NFKB [200]	17	6.24 × 10^−22^	4.78 × 10^−18^
Apoptotic process [1972]	25	3.18 × 10^−12^	1.22 × 10^−8^
Apoptotic signaling pathway [620]	14	2.33 × 10^−10^	5.96 × 10^−7^
Cellular response to organic substance [1992]	22	1.07 × 10^−9^	2.05 × 10^−6^
Regulation of intracellular signal transduction [1853]	21	1.74 × 10^−9^	2.66 × 10^−6^
Regulation of molecular function [1984]	20	3.17 × 10^−8^	4.05 × 10^−5^
Regulation of programmed cell death [1566]	17	1.4 × 10^−7^	1.07 × 10^−4^
Regulation of response to stress [1448]	16	2.7 × 10^−7^	1.72 × 10^−4^
Negative regulation of signaling [1520]	16	5.13 × 10^−7^	2.29 × 10^−4^
Cytokine-mediated signaling pathway [531]	10	5.31 × 10^−7^	2.29 × 10^−4^
Cytoskeleton organization [1525]	16	5.36 × 10^−7^	2.29 × 10^−4^

## Data Availability

Data sets are available for download at Havard Dataverse. https://doi.org/10.7910/DVN/RFNWTV (accessed on 3 June 2024).

## Supplementary Materials

The following supporting information can be downloaded at: https://www.mdpi.com/article/10.3390/ijms252312569/s1.
