# Supplementary material for: Molecular Composition and Kinetics of B Cells During Ibrutinib Treatment in Patients with Chronic Lymphocytic Leukemia
Source: Int J Mol Sci. 2024 Nov 22;25(23):12569. doi: 10.3390/ijms252312569 (PMC11641073; doi:10.3390/ijms252312569)
Supplement: Supplementary file 1 [file ijms-25-12569-s001.zip › ijms-3262196-supplementary.pdf]

Veyhe et al. 2024. Supplement. Part I

SUPPLEMENTAL METHODS, TABLES & FIGURES

Authors	
Abstract	Chronic lymphocytic leukemia (CLL) is characterized by the accumulation of B cells due to constitutive B-cell receptor (BCR) signaling, leading to apoptosis resistance and increased proliferation. This study evaluates the effects of Bruton Tyrosine Kinase (BTK) inhibitor ibrutinib on the molecu-lar composition, clonality, and kinetics of B cells during treatment in CLL patients. Employing a multi-omics approach of up to 3.2 years of follow-up, we analyzed data from 24 CLL patients, specifically focusing on nine patients treated with ibrutinib monotherapy. In this study, clonal stability was observed within the ibrutinib-treated group following an effective initial clinical response, where clonotype frequencies of residual CLL cells remained high and stable, ranging from 74.9% at 1.5 years to 87.7% at approximately 3 years. In contrast, patients treated with the B-cell lymphoma 2 (BCL2) inhibitor venetoclax exhibited substantial reductions in clonal frequencies, approaching molecular eradication. Deep whole-exome sequencing revealed minimal genomic progression in the ibrutinib group, maintaining somatic drivers and variant allele frequencies (VAF) above 0.2 throughout treatment. At the single-cell level, the NF-kB pathway inhibition and apoptotic signals were detected or even augmented during treatment in ibrutinib-treated patients. These findings may corroborate the role of ibrutinib in stabilizing the genomic landscape of CLL cells, preventing significant genomic evolution despite maintaining a high clonal burden within the residual B-cell compartment.
Compiled by	Marcus Høy Hansen, marcus.hoy.hansen@rsyd.dk

Supplementary methods

Patient Inclusion, Sample Storage and Processing

The characteristics and treatment details of the chronic lymphocytic leukemia (CLL) patient cohort, included over a 2.5-year period prior to receiving ibrutinib or venetoclax therapy, is outlined in **Table S1**. Study inclusion required informed consent and sufficient sample availability. Longitudinal clonotyping sample collection is depicted in **Figure S1**. The cohort comprised 24 patients (21 from Odense University Hospital and 3 from Aalborg University Hospital), with treatment assigned based on clinical guidelines and patient profiles. Nine patients received ibrutinib, while 15 underwent rituximab-venetoclax (R-v) or ibrutinib-venetoclax (categorized as venetoclax due to the therapeutic effects). Four venetoclax-treated patients had prior ibrutinib therapy.

Blood samples for LymphoTrack, exome, and transcriptome sequencing were processed on the same day or the next day and preserved in either DMSO or lysis buffer. Thirteen diagnostic samples were sourced from routine diagnostic procedures.

Table S1) Patient inclusion and profiles

Patient ID	Date of Diagnosis	Treatment Arm	Ibrutinib/R-v treatment start	Prior Treatments	Inclusion Date	Recruitment Type
620	Q3 2018	Ibrutinib	Q1 2020	1	Q3 2020	Routine care
623	Q4 2005	Ibrutinib	Q3 2019	0	Q3 2020	Routine care
664	Q4 2013	Ibrutinib	Q3 2019	1	Q4 2020	Routine care
692	Q4 2020	Ibrutinib	Q1 2021	0	Q4 2020	Routine care
705	Q1 2014	Ibrutinib	Q4 2019	2	Q1 2021	Routine care
875	Q2 2018	Ibrutinib	Q3 2021	2	Q4 2021	Routine care
911	Q3 2019	Ibrutinib	Q4 2021	2	Q4 2021	Routine care
1022	Q1 2022	Ibrutinib	Q2 2022	0	Q2 2022	Concurrent clinical study
1026	Q2 2017	Ibrutinib	Q2 2022	0	Q2 2022	Concurrent clinical study
252	Q2 2010	R-venetoclax	Q2 2021	3	Q4 2021	Routine care
294	Q1 2006	R-venetoclax	Q4 2021	2	Q4 2021	Routine care
647	Q2 2016	R-venetoclax	Q2 2020	1	Q4 2020	Routine care
670	Q1 2010	R-venetoclax	Q2 2020	1	Q4 2020	Routine care
714	Q4 2016	R-venetoclax	Q4 2020	1	Q1 2021	Routine care
741	Q3 2001	R-venetoclax	Q4 2020	1	Q1 2021	Routine care
748	Q3 2007	Ibrutinib-venetoclax	Q4 2018	1	Q3 2021	Concurrent clinical study
784	Q1 2014	R-venetoclax	Q2 2021	2	Q2 2021	Routine care
890	Q4 2010	R-venetoclax	Q4 2021	1	Q4 2021	Routine care
923	Q4 2013	R-venetoclax	Q3 2021	1	Q4 2021	Routine care
1057	Q3 2011	R-venetoclax	Q4 2022	1	Q2 2022	Routine care
1204	Q2 2012	R-venetoclax	Q1 2023	0	Q1 2023	Routine care
2626-1	Q1 2013	R-venetoclax	Q4 2021	3	Q4 2021	Concurrent clinical study
2626-2	Q3 2009	R-venetoclax	Q3 2022	1	Q3 2022	Concurrent clinical study
2626-5	Q1 2014	R-venetoclax	Q4 2022	1	Q4 2022	Concurrent clinical study

Figure S1) Treatment and sample overview

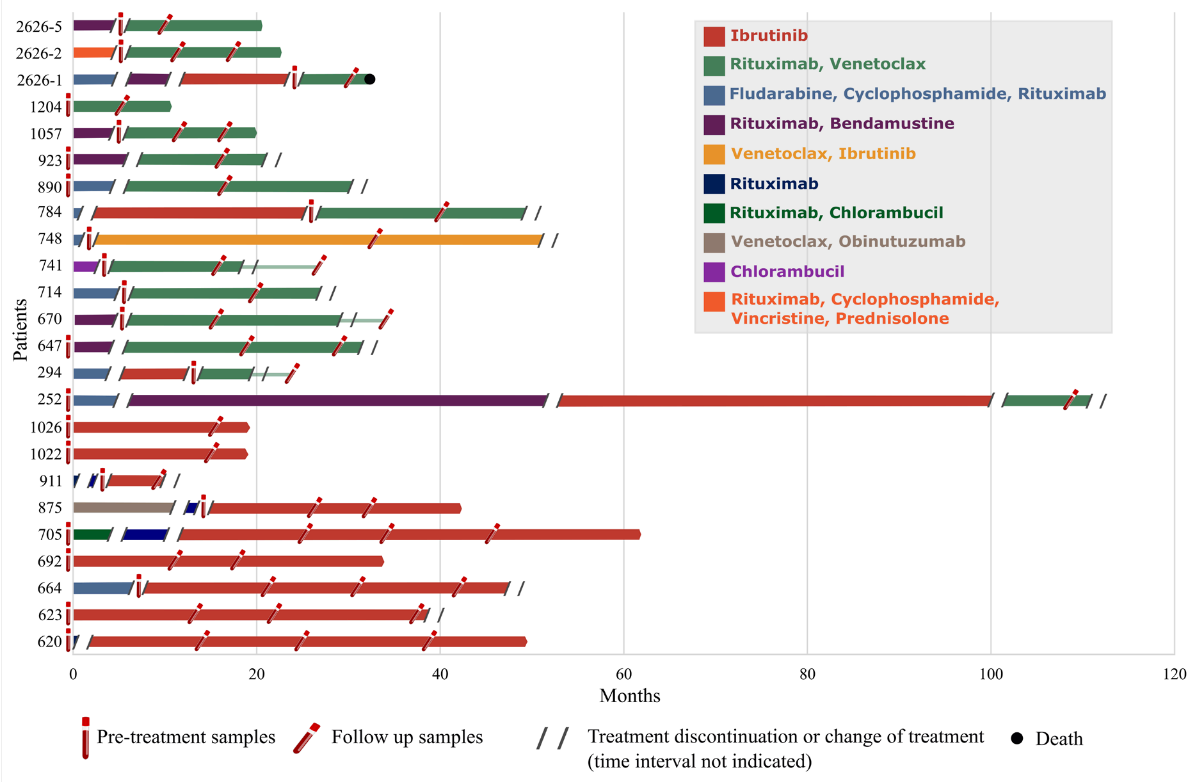

Figure S1 presents an overview of pre-treatment and follow-up samples included in the IGH clonality analysis, along with retrospective time points. The X-axis presents the duration of the different treatment lines. Wait and watch periods are not depicted in the figure. Discontinuation or change of treatment is indicated by / /, whereas continuation of treatment is shown with an arrow.

Flow Cytometry Analysis Using the LST Panel

We analyzed 37 samples using the LST panel for flow cytometry (**Table S2**). The median duration from sample collection to staining and analysis was two days, with a range from 0 to 7 days. For samples older than three days, comparisons were made with the purity profiles of newer samples or samples from the same period. Staining with the LST panel was either not feasible or insufficient for thirteen samples. Hence, the percentage of CD19+ cells was determined using PBMCs stained with the purity panel. CD19+ percentages below 0.1 were evaluated as non-detectable (ND) due to detection limit uncertainties.

Table S2) Flow cytometry LST panel

Antigen	Fluorophore	Company	Clone
CD8+Lambda<br>CD56+Kappa	FITC<br>PE	Cytognos	UCHT-4<br>C5.9
CD5	PerCP-Cy5.5	BD	L17F12
CD3	APC	BD	SK7
CD38	APC-H7	BD	HB7
CD4	Pacific blue (PB)	BioLegend	RPA-T4
CD20	Pacific blue (PB)	BioLegend	2H7
CD45	Pacific Orange (PO)	ThermoFisher	HI30
CD19	PE-Cy7	Beckman Coulter	J3-119
TCRγδ	PE-Cy7	BD	11F2

Cell Isolation and Flow Cytometry Procedures

Peripheral Blood Mononuclear Cells (PBMCs) were stored in a solution of 50% FBS (Gibco, part of Thermo Fisher Scientific, Waltham, MA, USA), 30% RPMI medium (Gibco, Thermo Fisher Scientific), and 10% DMSO (Sigma-Aldrich, St. Louis, MI, USA). Upon thawing, cells were added to pre-warmed Hank's Balanced Salt Solution (HBSS; Gibco, Thermo Fisher Scientific) supplemented with 20% FBS, 2 mM $MgCl_2$ (Sigma-Aldrich), and 50 µg/ml DNase I (Roche, Basel, Switzerland). Cells were then washed and resuspended in HBSS with 2% FBS, 50 µg/ml DNase I, and 2 mM $MgCl_2$, targeting a concentration of 1 million cells per ml.

Cells were sorted using either OctoMACS with MS columns or QuadroMACS with LS columns (Miltenyi Biotec, Bergisch Gladbach, Germany), applying negative enrichment via the Pan B Cell Isolation Kit or the Pan T Cell Isolation Kit (Miltenyi Biotec). Cell concentrations were determined manually using counting chambers stained with trypan blue (Sigma-Aldrich) or measured on a NucleoCounter NC-202 using Via2-Cassettes (ChemoMetec, Allerød, Denmark). Prior to and following isolation, flow cytometry was employed to assess the purity of isolated B and T cells using BD FACSLyric and BD FACSCanto systems (BD Biosciences, Franklin Lakes, NJ, USA). Analyses were performed with FlowLogic Software (Inivai Technologies, Mentone Victoria, Australia) or FCS Express Software (De Novo Software, Pasadena, CA, USA). Cells were stained with a series of conjugated antibodies including **(Table S3)**.

Table S3) Antibodies used for staining

Marker	Fluorochrome	Clone	Company
Live/dead marker	APC-H7		Invitrogen
CD45	PO	MHCD4530	Life technologies
CD3	APC	SK7	BD
CD56	PE	C5.9	Cytognos
CD19	PE-Cy7	Cy7 J3-11	Beckman Coulter
CD20	PB	2H7	Biolegend
Kappa/lambda	AF488		Biolegend
CD5	PerCP-Cy5.5	L17F12	BD

Table S4A presents the IGH clonotype burden at several treatment stages: pre-treatment, 0–0.5 years, 0.5–1.5 years, and 1.5–2.5 years. It shows the frequency of clonal cells relative to total rearranged B cells, clonal counts, and the total number of reads analyzed. The repertoire of variable (V), diversity (D), and joining (J) gene segments, verified using NCBI IgBLAST [Pubmed ID: 23671333], is also listed.

Table S4A) IGH clonality analysis for ibrutinib treated patients

Pt ID		Pre-treatment	0.5–1.5 yrs	1.5–2.5 yrs	2.5–3.2 yrs	V gene	D gene	J gene
**620**	Clonal frequency (Clonal count)	91.84% (705079)	89.98% (641381)	89.96% (468445)	89.41% (538326)	V1-3*01	D6-19*01	J4*02
	Total read analyzed	767741	712837	520.700	602.110			
**623**	Clonal frequency (Clonal count)	73.72% (526361)	69.52% (486899)	88.38% (657732)	88.13% (517992)	V3-30*04	D3-9*01	J4*02
	Total read analyzed	714024	700378	744238	587748			
**664**	Clonal frequency (Clonal count)	88.40% (824773)	74.96% (3630011)	82.72% (527184)	87.34% (527343)	V1-69*06	D6-19*01	J6*02
	Total read analyzed	933038	4842443	637.344	603774			
**692**	Clonal frequency (Clonal count)	78.43% (182223)	12.77% (95333)	15.38% (105724)	-	V1-69*01	D3-16*03	J3*02
	Total read analyzed	232341	746750	687518	-			
**705**	Clonal frequency (Clonal count)	86.38% (207205)	83.54% (695963)	82.99% (577803)	85.17% (536283)	V3-30*18	D6-19*01	J4*02
	Total read analyzed	239875	833.075	696.273	629.629			
**875**	Clonal frequency (Clonal count)	84.73% (575369)	85.14% (256704)	81.25% (370814)	-	V3-9*01	D6-13*01	J4*02
	Total read analyzed	679063	301526	456369	-			
**911**	Clonal frequency (Clonal count)	65.35% (283135)	67.88% (279646)	-	-	V3-22*01	D2-21*02	J4*02
	Total read analyzed	433263	411991	-	-			
**1022**	Clonal frequency (Clonal count)	54.80% (296838)	66.81% (404175)	-	-	V3-23*01	D3-22*01	J4*02
	Total read analyzed	541643	604970	-	-			
**1026**	Clonal frequency (Clonal count)	86.41% (652949)	88.61% (514436)	-	-	V1-69*01	D3-3*01	J6*02
	Total read analyzed	755622	580586	-	-			

Table S4B presents the IGH clonotype burden at several treatment stages: pre-treatment, 0–0.5 years, 0.5–1.5 years, and 1.5–2.5 years. It informs the frequency of clonal cells relative to total rearranged B cells, clonal counts and the total number of reads analyzed. Additionally, the table includes the estimated cell equivalent (ECE) or the detection confidence at 10^{-4}, where available. The repertoire of variable (V), diversity (D), and joining (J) gene segments, verified using NCBI IgBLAST [Pubmed ID: 23671333], is also displayed.

Table S4B) IGH clonality analysis for Venetoclax-treated patients (ND: non-detectable, NA: not available)

Pt ID		Pre- treatment	0–0.5 yrs	0.5–1.5 yrs	1.5–2.5 yrs	V gene	D gene	J gene
**252**	Clonal frequency (Clonal count)	82.89% (348227)	-	82.99% (693628)	-	V4-30-4*09	D2-15*01	J4*02
	ECE	-	-	4206	-			
	Total read analyzed	420118	-	835829	-			
**294**	Clonal frequency (Clonal count)	85.80% (953605)	-	-	-	V3-49*04	D3-10*01	J6*03
	ECE	-	-	ND	-			
	Confidence at 10^{-4}	-	-	98.63%	-			
	Total read analyzed	1111440	-	350960	-			
**647**	Clonal frequency (Clonal count)	24.36% (114095)	-	-	0.02% (247)	V1-2*02	D6-19*01	J5*02
	ECE	-	-	ND	0.75			
	Confidence at 10^{-4}	-	-	99.13%	-			
	Toal read analyzed	468359	-	284698	1447241			
**670**	Clonal frequency (Clonal count)	48.72% (470490)	-	2.24% (21500)	0.46% (3178)	V1-8*01	D6-6*01	J4*02
	ECE	-	-	14.62	-			
	Total read analyzed	965.664		959701	684217			
**714**	Clonal frequency (Clonal count)	82.57% (179074)	-	19.74% (252442)	-	V3-48*03	D1-1*01	J4*02
	ECE	-	-	169	-			
	Total read analyzed	216883	-	1278642	-			
**741**	Clonal frequency (Clonal count)	51.05% (529344)	-	-	-	V1-3*04	D3-10*01	J4*02
	ECE	-	-	ND	ND			
	Confidence at 10^{-4}	-	-	99.81%	95.26%			
	Total read analyzed	1036943	-	438422	506109			
**784**	Clonal frequency (Clonal count)	82.75% (120230)	-	17.61% (18194)	-	V3-23*01	D1-26*01	J4*02
	ECE	-	-	-	-			
	Total read analyzed	145299	-	103.310	-			

**890**	Clonal frequency (Clonal count)	51.29% (237519)	-	-	-	V3-23*01	D3-22*01	J4*02
	ECE	-	-	ND	-			
	Confidence at 10^{-4}	-	-	98.61%	-			
	Total read analyzed	463074	-	325165	-			
**923**	Clonal frequency (Clonal count)	66.02% (92016)	-	0.34% (1457)	-	V3-30*04	D2-15*01	J6*02
	ECE	-	-	1.61	-			
	Confidence at 10^{-4}	-	-	-	-			
	Total read analyzed	139384	-	424595	-			
**1057**	Clonal frequency (Clonal count)	84.80% (676384)	0.01% (71)	1.73% (8)	-	V4-38-2*02	D6-19*01	J4*02
	ECE	-	0.05	NA	-			
	Confidence at 10^{-4}	-	-	-	-			
	Total read analyzed	797597	704982	463	-			
**1204**	Clonal frequency (Clonal count)	74.56% (379499)	17.60% (47)	-	-	V1-18*01	D3-3*01	J6*02
	ECE	-	-	-	-			
	Total read analyzed	508962	267	-	-			
**2626-1**	Clonal frequency (Clonal count)	90.56% (599772)	-	0.44% (3852)	-	V3-21*01	N/A	J6*02
	ECE	-	-	4.47	-			
	Total read analyzed	662294	-	883298	-			
**2626-2**	Clonal frequency (Clonal count)	73.85% (401839)	0.01% (10)	0.002% (19)	-	V4-61*12	D3-16*01	J5*02
	ECE	-	0.03	0.04	-			
	Total read analyzed	544.131	110.598	918.823	-			
**2626-5**	Clonal frequency (Clonal count)	90.58% (506315)	-	-	-	V3-64D*06	D3-10*01	J4*02
	ECE	-	ND	-	-			
	Confidence at 10^{-4}	-	99.32%	-	-			
	Total read analyzed	558992	380999	-	-			
**748**	**Months**	**-**	**-**	**-**	**32**	V3-48*02	D3-22*01	J3*02
	Clonal frequency (Clonal count)	85.79% (483736)	-	-	15.0% (168050)			
	Total read analyzed	563873	-	-	1120344			

Table S5A presents lymphocyte counts (measured in 10^9/L) for ibrutinib-treated patients. The counts are obtained using the Sysmex XN-series or XR-series analyzers (Sysmex Corporation). It also includes the percentage of CD19-positive B cells obtained by flow cytometry, determined from the lymphocyte gate **(see gating strategy, Figure S2)**, and a rough estimate of CLL cell counts. The estimated CLL cell counts are calculated by multiplying the total lymphocyte count by the percentage of CD19+ cells and the burden of IGH clonotypes.

Table S5A) Lymphocyte and CLL cell counts in Ibrutinib-treated patients

	Lymphocyte count (10^9/L)				CD19 positive cells from lymphocytes (%)				Estimated count of clonal cells (10^9/L)			
**ID**	**Pre-treatment**	**0.5–1.5 yrs**	**1.5–2.5 yrs**	**2.5–3.2 yrs**	**Pre-treatment**	**0.5–1.5 yrs**	**1.5–2.5 yrs**	**2.5–3.2 yrs**	**Pre-treatment**	**0.5–1.5 yrs**	**1.5–2.5 yrs**	**2.5–3.2 yrs**
**620**	51.50	2.90	2.15	1.61	-	19.5	21.06	21.9	42.5	0.5	0.4	0.3
**623**	5.15	13.5	10.20	6.56	-	31.3	31.3	11.4	3.4	2.9	2.8	0.7
**664**	54.9	7.23	2.14	1.15	-	86.7	63.69	24.7	43.6	4.7	1.1	0.2
**692**	6.95	2.89	2.78	-	73.4	1.2	0.52		4.9	4.6E-3	2.2E-03	-
**705**	37.1	2.98	3.63	2.05	-	25.8	37.52	27.4	28.8	0.6	1.1	0.5
**875**	14.3	2.60	2.79	-	-	24.1	2.43	-	10.9	0.5	0.1	-
**911**	2.05	0.70	-	-	76.4	55.8	-	-	1.2	0.3	-	-
**1022**	127.00	8.87	-	-	90.0	61.5	-	-	62.5	3.6	-	-
**1026**	20.90	3.66	-	-	90.0	35.6	-	-	16.2	1.2	-	-

Table S5B presents lymphocyte counts (measured in 10^9/L) for venetoclax-treated patients. Counts were obtained using the Sysmex XN-series or XR-series analyzers (Sysmex Corporation). It also includes the percentage of CD19-positive B cells obtained by flow cytometry, determined from the lymphocyte gate, and a rough estimate of CLL cell counts. The estimated CLL cell counts are calculated by multiplying the total lymphocyte count by the percentage of CD19+ cells and the burden of IGH clonotypes. Samples with counts below the detection limit are presented as non-detectable (ND) and samples below the detection limit given by IGH sequencing are marked with an asterisk (*).

Table S5B) Lymphocyte and CLL cell counts in venetoclax-treated patients

	Lymphocyte count (10^9/L)					CD19 positive cells from lymphocytes (%)					Estimated count of clonal cells (10^9/L)				
**ID**	**Pre-treatment**	**0–0.5 yrs**	**0.5–1.5 yrs**	**1.5–2.5 yrs**	**2.5–3.2 yrs**	**Pre-treatment**	**0–0.5 yrs**	**0.5–1.5 yrs**	**1.5–2.5 yrs**	**2.5–3.2 yrs**	**Pre-treatmen**	**0–0.5 yrs**	**0.5–1.5 yrs**	**1.5–2.5 yrs**	**2.5–3.2 yrs**
**252**	7.49	-	0.46	-	-	-	-	17.6	-	-	5.6	-	$6.7 \cdot 10^{-2}$	-	-
**294**	34.50	-	1.78	-	-	93.0		ND*	-	-	26.6	-	ND*	-	-
**647**	1.11	-	0.84	2.48	-	-	-	ND	0.2	-	0.2	-	ND*	$3.60 \cdot 10^{-6}$	-
**670**	68.00	-	0.37	0.61	-	-	-	ND	9.2	-	29.8	-	ND	$2.6 \cdot 10^{-4}$	-
**714**	14.30	-	0.95		-	-	-	ND	-	-	10.6	-	ND	-	-
**741**	152.00	-	0.39	0.70	-	-	-	ND	18.2	-	69.7	-	ND*	ND*	-
**784**	59.90	-	1.02	-	-	95.0	-	1.8	-	-	44.5	-	$3.2 \cdot 10^{-3}$	-	-
**890**	41.80	-	0.97	-	-	96.0	-	ND*	-	-	19.3	-	ND*	-	-
**923**	131.00	-	0.95	-	-		-	ND	-	-	77.7	-	ND	-	-
**1057**	144.00	5.78	7.66	-	-	74.1	ND*	ND	-	-	109.7	ND	ND	-	-
**1204**	172.00	0.56		-	-	95.1	21.0	-	-	-	115.2	$2.1 \cdot 10^{-2}$	-	-	-
**2626-1**	7.97		0.28	-	-	89.7		-	-	-	6.5	-	-	-	-
**2626-2**	46.18	1.14	1.06	-	-	88.1	1.1	ND	-	-	30.6	$1.3 \cdot 10^{-6}$	ND	-	-
**2626-5**	21.51	1.70	-	-	-	85.3	ND	-	-	-	17.5	ND*	-	-	-
**748**	38.40	-	-	-	1.30	-	-	-	-	0.98	-	-	-	-	19.1

Figure S2) Gating strategy for evaluating CD19-positive cells

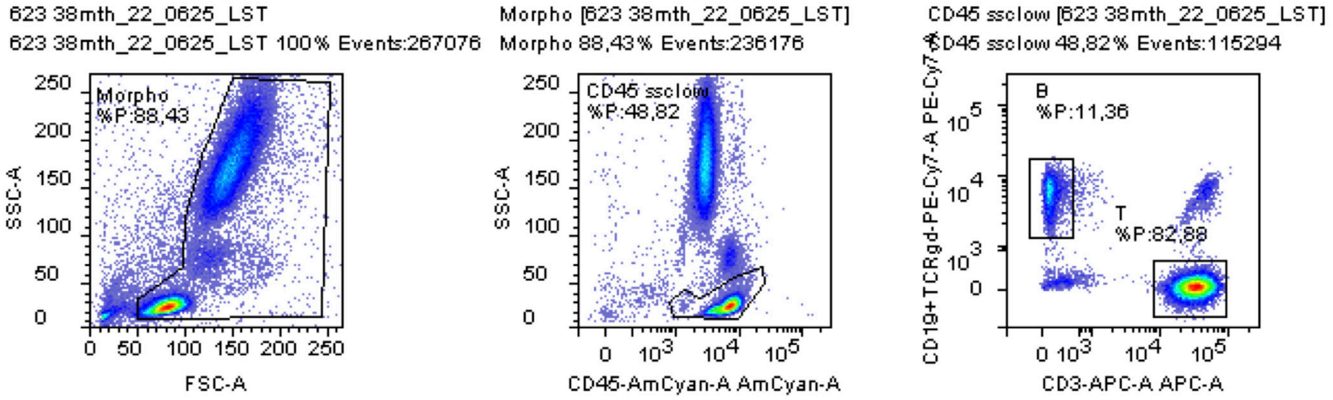

Veyhe et al. 2024. Supplement. Part II

DEEP WHOLE-EXOME SEQUENCING OF CLL SAMPLES

Number of samples sequenced	49
Sequencing details	Sequenced on NovaSeq 6000, S4 flow cell in 2 runs (standard run S4 310 µl, protocol: TWIST Mechanical Fragmentation and Twist Universal adapter system and TWIST target enrichment protocol (Twist Bioscience). Adaptor from IDT: xGen UDI-UMI Adapters, 1-96. (PN: 10006913). 15 µM/well. Amplicon pools were diluted to 4 nM in a volume of 50 µl: **Run #1** amplicon pool A, B, C conc. of 28.78, 30.54, 35.75 nM: 6.95, 6.55, and 5.59 µl to 43.05, 43.45, and 44.41 µl H_2O. **Run #2** amplicon pool A, B, C, D conc. of 77.29, 49.12, 67.39 77.29 nM: 2.59, 4.07, 2.97, 2.59 µl to 47.41, 45.93, 47.03, 47.41 µl H_2O.<br><br>Loading: PhiX 1 µl + 3 µl EB. NaOH: 10 µl 2N NaOH + 90 µl H_2O. 8 min incubation and 40 µl Tris + 60 µl H_2O.

**Pool**	**A**	**B**	**C**	
**Sequencing run #1**				
conc. after hybridi-zation ng/ul	7.56	8.02	9.39	-
Fragment length	400	400	400	-
Amplicon pool conc. nM	28.78	30.54	35.75	-
Diluted to 4 nM				
Pool µl	6.95	6.55	5.59	-
H_2O µl	43.05	43.45	44.41	-
Total µl	50.00	50.00	50.00	-
**Sequencing run #2**				
**Pool**	**A**	**B**	**C**	**D**
conc. after hybridi-zation ng/ul	20.3	12.9	17.7	20.3
Fragmentlængde	400	400	400	400
Amplicon pool conc. nM	77.29	49.12	67.39	77.29
Diluted to 4 nM				
Pool µl	2.59	4.07	2.97	2.59
H_2O µl	47.41	45.93	47.03	47.41
Total µl	50.00	50.00	50.00	50.00

Compiled by	Marcus Høy Hansen, marcus.hoy.hansen@rsyd.dk

Sample cohort

A total of 49 samples, including 11 T-cell control counterparts, were prepared for whole-exome sequencing. Blood samples were collected at diagnosis or before the initiation of treatment, with follow-up samples obtained subsequently, spanning 1 to 5 samples across a period of 0 to 38 months. Cryopreserved cells were not available for 10 diagnostic samples. Instead, PBMCs were used, yielding 7061 ng (846–12950 ng) for these samples. All other 28 samples consisted of purified cells. Isolated B cells had a median DNA yield of 1575 ng (45–16100 ng) with a purity of 87.0% (77.9%–94.1%) and a T-cell infiltration of 0.02% (0–0.5%), measures by flow cytometry. T cells yielded a median DNA amount of 1371 ng (326–25760 ng) with a purity of 72.6% (31.1%–86.7%) and a B-cell infiltration of 0.4% (0–6.2%). For the purified B cell samples, the flow cytometry

assessments showed a viability of 92.41% (84.14–97.83%). While the counted viability measured by trypan blue was 98.6% (90–100%). T cell viability assessed by flow cytometry was 87.6% (67.4–96.6%), while the viability using trypan blue was 93.0% (81.5–97.8%).

Sequencing libraries were prepared using the Twist Exome 2.0 Hybridization and Target Enrichment Kit (Twist Bioscience, South San Francisco, CA, USA), which covers 36.5 Mb of protein-coding regions. Sequencing was conducted in two separate sequencing runs on the NovaSeq 6000 System (Illumina) using 2 x 151 cycle paired-end sequencing on S4 flow cells **(Listing 1)**.

Listing 1) Sequencing run layouts

Sequencing run #1

Pool	#	Patient ID	Sample	Adaptor
A	1	705	0020	A1
	2	923	0015	B1
	3	623	0017	C1
	4	911	0018	D1
	5	620	0024	E1
	6	664	0025	F1
	7	670	0032	G1
	8	692	0035	H1
B	9	875	0034	A2
	10	741	0033	B2
	11	748	0042	C2
	12	923	0043	D2
	13	705	0074	E2
	14	923	0075	F2
	15	623	0084	G2
	16	911	0085	H2
C	17	620	0086	A10
	18	664	0098	B10
	19	670	0099	D10
	20	741	0100	E10
	21	875	0136	F10
	22	692	0135	G10
	23	748	0142	H10

Sequencing run #2

Pool	#	Patient ID	Sample	Adaptor
A	1	623	0384	A1
	2	692	0412	B1
	3	705	0413	C1
	4	620	0414	D1
	5	623	0421	E1
	6	664	0422	F1
B	7	911	0428	G1
	8	923	0430	H1
	9	705	0443	A2
	10	692	0444	B2
	11	692	0445	C2
	12	623	0446	D2

C	13	664	0447	E2
	14	620	0448	F2
	15	620	0449	G2
	16	875	0455	H2
	17	664	0566	A3
	18	620	0567	B3
	19	623	0569	C3
D	20	705	0336	D3
	21	741	0076	E3
	22	670	0077	F3
	23	748	0078	G3
	24	911	0441	H3
	25	875	0442	A4
	26	923	0457	B4

Bioinformatics summary

Sequenced data were aligned to the reference genome (GRCh38/Hg38, GATK Resource Bundle) using BWA ([PMID: 20080505], (bwa mem v0.7.17-6) on the UCloud interactive HPC system (University of Southern Denmark, eScience Center, DK), running 63 threads per paired-end alignment on Ubuntu 22.04 with 64 vCPU and 384 GB RAM confined to target regions (Twist Human Core Exome, Twist Bioscience; samtools view, v1.13 [19505943]). Duplicate reads were marked using MarkDuplicatesSpark (GATK v4.3.0.0) on Ubuntu 18.04 for parallel processing. Nucleotide variants were called from base quality score recalibrated alignments (BaseRecalibrator and ApplyBQSR, GATK) using control-paired samples (Mutect2, FilterMutectCalls) or for quality assessment of single-nucleotide variant allele frequencies (VAF) [PMID: 36283582] (HaplotypeCaller, SelectVariants, VariantRecalibrator, ApplyVQSR) and detection of allelic imbalance. Variants covered less than 100x were discarded (VariantFiltration). Downstream somatic variant annotation and filtration was performed using SnpEff (GRCh38.p13, dbNSFP v4.1a) [PMID: 22728672] and SNPsift (dbNSFP v4.1a) [PMID: 22435069] with external data sources gnomAD (v3.1.2) [32461654], ClinVar (Dec 11, 2022) [PMID: 29165669], COSMIC (v.98 [PMID: 30371878]).

Coverage profiles from BEDtools [20110278]) and VAF were compared for the combined assessment of chromosomal CNAs and allelic imbalances and analyzed using Wolfram Mathematica (v12, Wolfram Research, Champaign, Illinois, USA).

The bioinformatics pipeline partly adopted the Broad Institute GATK Best Practices Workflow (GATK 4.3.0.0) focusing on short variant discovery from DNA (Listing 2). Marking of duplicates was performed. Deviations included single-sample calling of variants for quality assessment and copy-number analyses. Mutect2 was implemented for the detection of low allele-frequency variants, tolerating some contamination of B cells in T-cell control samples. Because of this, no hard filtering was used, only flagging of germline contamination.

Listing 2) The workflow was adapted from Broad Institute's Best Practices workflow on DNA short variant discovery

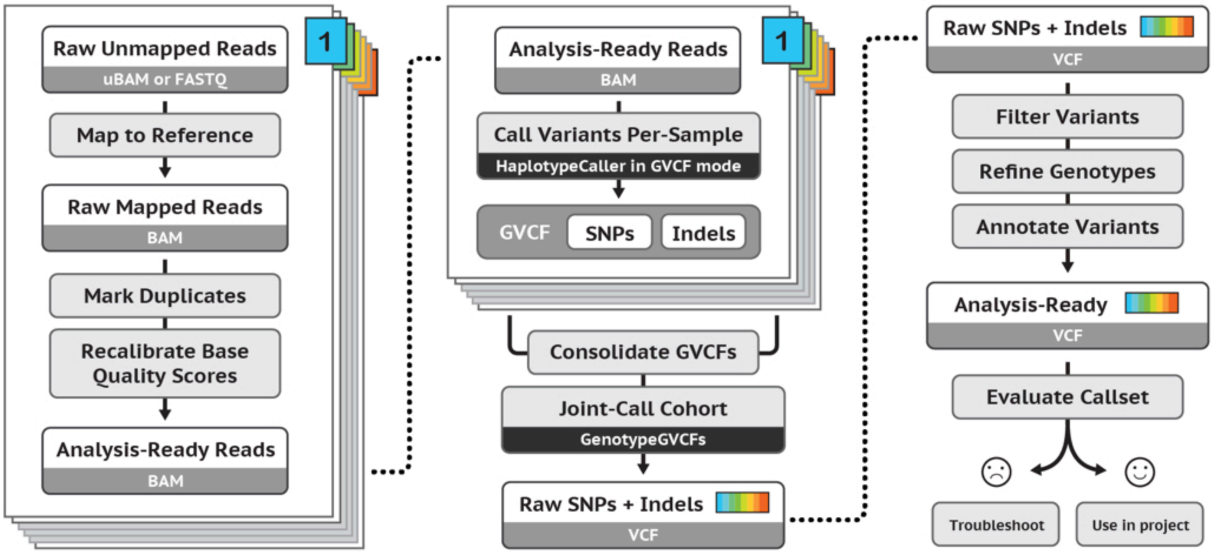

source: gatk.broadinstitute.org, short variant discovery (SNPs + Indels), accessed January 2024

Initial quality assesment was performed using fastqc (**Listing 3**) and showed a general high quality as shown in example (**Listing 4**).

Listing 3) Running parallel fastqc

```
sudo apt-get update
sudo apt-get install fastqc
fastqc *_trim_R*_001.fastq.gz -t 31
```

Listing 4) FASTQC example output

Measure	Value
Filename	G147-23-0017_twist-exome_HKN3FDSX5_R1.fastq.gz
File type	Conventional base calls
Encoding	Sanger / Illumina 1.9
Total Sequences	573659740
Sequences flagged as poor quality	0
Sequence length	151
%GC	48

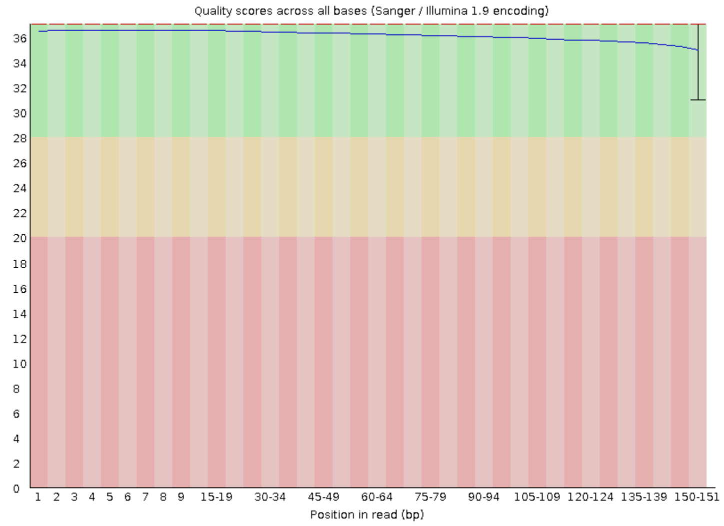

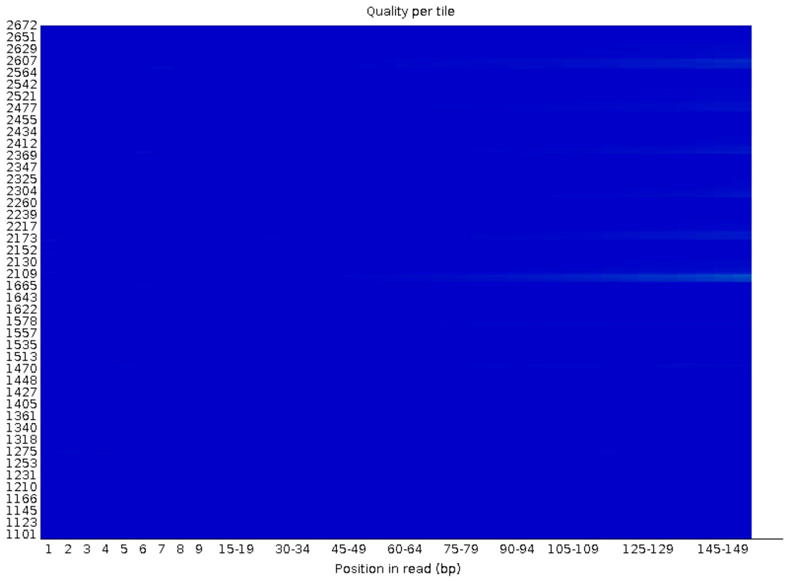
Quality per tile
Position in read (bp)

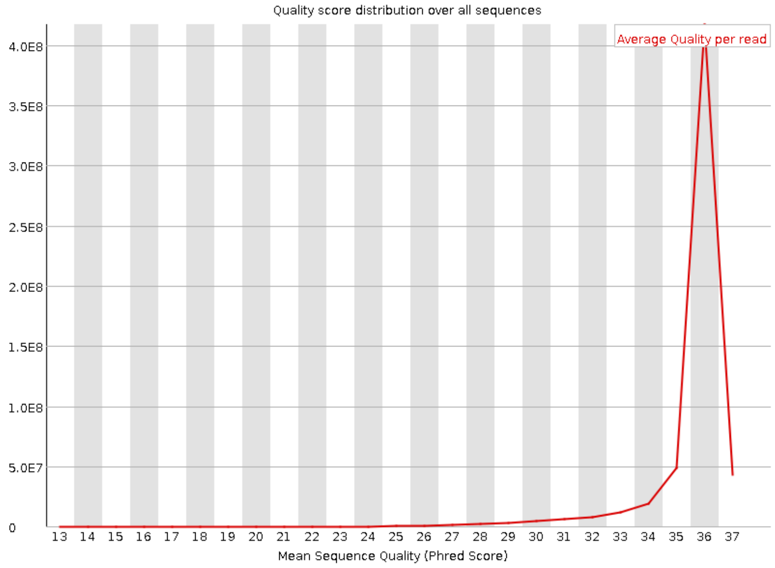
Quality score distribution over all sequences
Average Quality per read
Mean Sequence Quality (Phred Score)

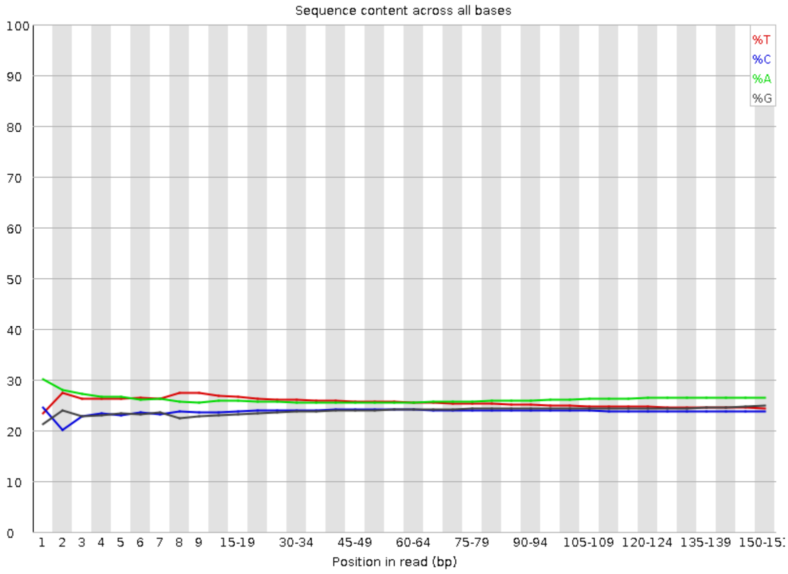
Sequence content across all bases
%T
%C
%A
%G
Position in read (bp)

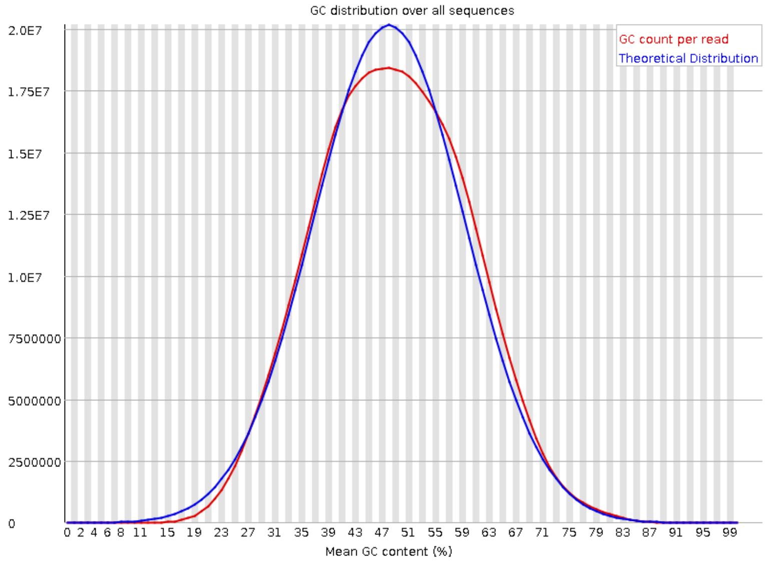
GC distribution over all sequences
GC count per read
Theoretical Distribution
2.0E7
1.75E7
1.5E7
1.25E7
1.0E7
7500000
5000000
2500000
0
0 2 4 6 8 11 15 19 23 27 31 35 39 43 47 51 55 59 63 67 71 75 79 83 87 91 95 99
Mean GC content (%)

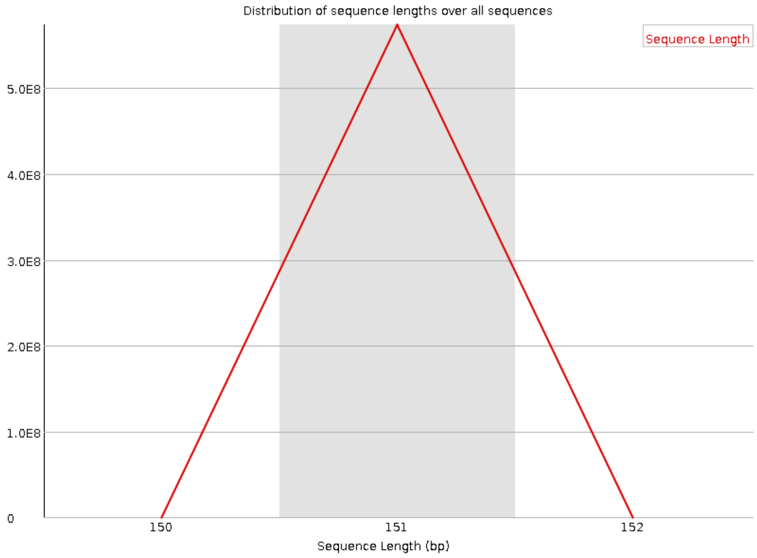
Distribution of sequence lengths over all sequences
Sequence Length
5.0E8
4.0E8
3.0E8
2.0E8
1.0E8
0
150
151
152
Sequence Length (bp)

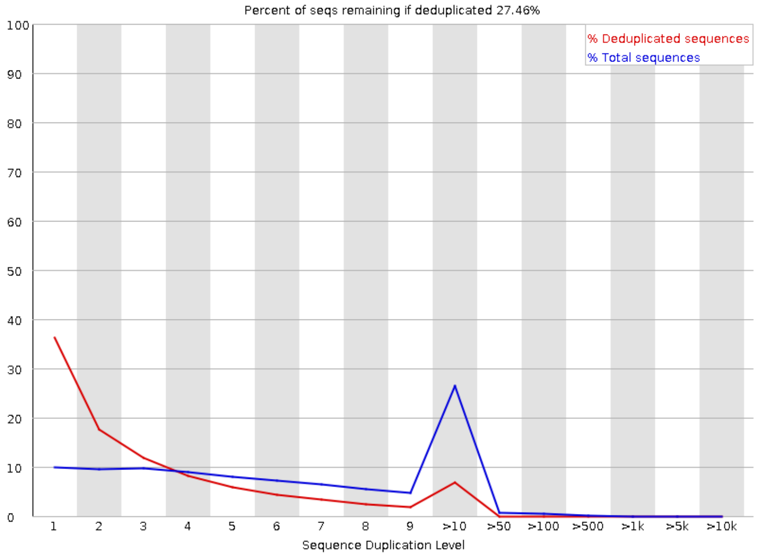
Percent of seqs remaining if deduplicated 27.46%
% Deduplicated sequences
% Total sequences
100
90
80
70
60
50
40
30
20
10
0
1 2 3 4 5 6 7 8 9 >10 >50 >100 >500 >1k >5k >10k
Sequence Duplication Level

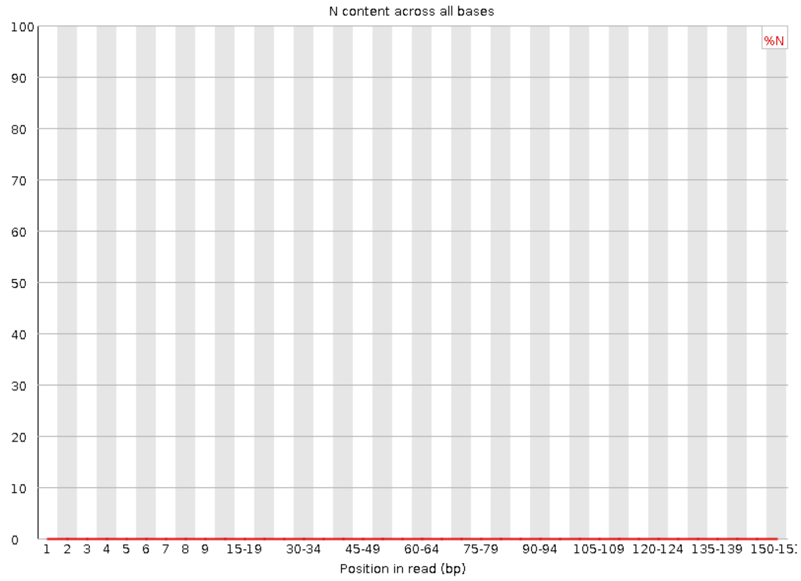

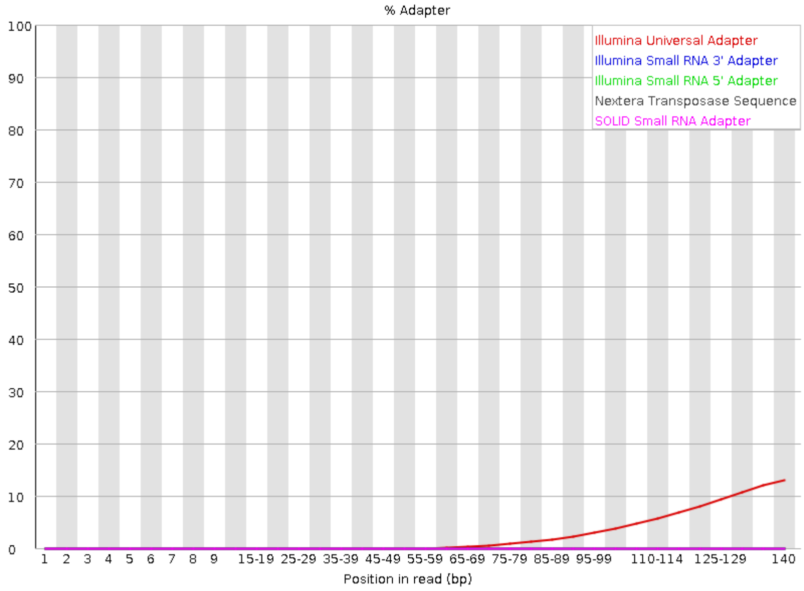

FASTQ files were trimmed using fastp, wrapped in a bash-script (Listing 5) and parallelized using mutip-le processes. The trimming script was invoked for all FASTQ files in folders with sample ID as parameter. (Listing 6).

Listing 5) Trimming whole-exome sequencing reads

```
#!/bin/bash

fn=$1

#sudo apt-get update
#sudo apt-get install fastp
# Usage: for f in *_L001_R1_001.fastq.gz; do (./fastp_trim.sh ${f/_L001_R1_001.fastq.gz/})&; done


fastp -i $fn’_L001_R1_001.fastq.gz’ -I $fn’_L001_R2_001.fastq.gz’ -o $fn’_trim_R1_001.fastq.gz’ -O $fn’_
trim_R2_001.fastq.gz’ -h $fn’_fastp.html’ -j $fn’_fastp.json’ -w 5 --trim_tail1 5 -l 80 --trim_front1 5
```

file name: fastp_trim.sh

Listing 6) Running parallel trimming

```
for f in *_L001_R1_001.fastq.gz; do (./fastp_trim.sh ${f/_L001_R1_001.fastq.gz/})&; done
```

Alignment was performed on trimmed reads using a 32 vCPU machine (Listing 7). Targets were confined to the panel footprint.

Listing 7) Performing alignment on trimmed sequences

```
#!/bin/bash
set -x

file=$1

cd /ucloud/
cp '/work/CLL/fastqs/'$file'_trim_R1_001.fastq.gz' .
cp '/work/CLL/fastqs/'$file'_trim_R2_001.fastq.gz' .
cp -R /work/CLL/hg38 .
cp /work/CLL/Twist_Exome_Core_Covered_Targets_hg38.bed .

threads=31

sudo apt update
sudo apt install bwa
sudo apt -y install samtools

bwa mem -M -R "@RG\tID:group1\tSM:$1\tPL:illumina\tLB:lib1\tPU:unit1" -t $threads hg38/resources_broad_hg38_
v0_Homo_sapiens_assembly38.fasta $file"_trim_R1_001.fastq.gz" $file"_trim_R2_001.fastq.gz" | samtools view -L
Twist_Exome_Core_Covered_Targets_hg38.bed -@ $threads -m 4G -Sb -> $file.bam

mv $file'.bam' /work/CLL/WESBAM/.

rm $file'_trim_R1_001.fastq.gz'
rm $file'_trim_R2_001.fastq.gz'
rm -r hg38
rm Twist_Exome_Core_Covered_Targets_hg38.bed


# Run for each file using this run.sh script
```

run.sh

Marking of sequencing duplicates was done with GATK's MarkDuplicates implementing Spark for performance (MarkDuplicatesSpark) (Listing 8).

Listing 8) Marking of duplicates

```
#!/bin/bash
set -x

fn=$1

sudo apt update
sudo apt -y install samtools

cd /ucloud/
cp '/work/CLL/WESBAM/'$fn'.bam' .
cp /work/CLL/gatk-4.3.0.0.zip .
unzip -o gatk-4.3.0.0.zip

gatk-4.3.0.0/gatk MarkDuplicatesSpark -I $fn'.bam' -O $fn'.dedup.bam'
samtools index $fn'.dedup.bam' -@ 31
cp $fn'.dedup.bam' /work/CLL/WESBAM/.
cp $fn'.dedup.bam.bai' /work/CLL/WESBAM/.
cp $fn'.dedup.bam.sbi' /work/CLL/WESBAM/.

rm $fn'.bam'
rm $fn'.dedup.bam'

# Called for each of the files
#./markduplicates.sh filename
```

md.sh

Quality score recalibration was done using GATK's BaseRecalibrator using a 8 vCPU (Listing 9) and the recalibrated BAM were assessed using samtools stats and flagstat and summarised using Mathematica (see (Listing 10), (Listing 11), (Listing 12), (Listing 13)).

Listing 9) Performing recalibration

```
#!/bin/bash
set -x

fn=$1

sudo apt update
sudo apt -y install samtools

cd /ucloud/
cp '/work/CLL/WESBAM/'$fn'.dedup.bam' .
cp -R /work/CLL/hg38 .
cp /work/CLL/Twist_Exome_Core_Covered_Targets_hg38.bed .
cp /work/CLL/gatk-4.3.0.0.zip .
unzip -o gatk-4.3.0.0.zip

samtools index $fn'.dedup.bam' -@8

gatk-4.3.0.0/gatk BaseRecalibrator \
-I $fn'.dedup.bam' \
-R hg38/resources_broad_hg38_v0_Homo_sapiens_assembly38.fasta \
-L Twist_Exome_Core_Covered_Targets_hg38.bed \
--known-sites hg38/resources_broad_hg38_v0_1000G_phase1.snps.high_confidence.hg38.vcf.gz \
--known-sites hg38/resources_broad_hg38_v0_Homo_sapiens_assembly38.dbsnp138.vcf \
--known-sites hg38/resources_broad_hg38_v0_Homo_sapiens_assembly38.known_indels.vcf.gz \
--known-sites hg38/resources_broad_hg38_v0_Mills_and_1000G_gold_standard.indels.hg38.vcf.gz \
-O $fn.recal.table

gatk-4.3.0.0/gatk ApplyBQSR \
-I $fn.dedup.bam \
-R hg38/resources_broad_hg38_v0_Homo_sapiens_assembly38.fasta \
-L Twist_Exome_Core_Covered_Targets_hg38.bed \
--bqsr-recal-file $fn.recal.table \
-O $fn.dedup.recal.bam

cp $fn.dedup.recal.bam /work/CLL/WESBAM/.
rm $fn.dedup.recal.bam
rm $fn.dedup.bam
rm fn.recal.table
```

(recalibrate.sh)

Listing 10) Getting general read statistics using samtools stats

```
# Assuming that samtools is installed

# Get read statistics for Mathematica and save the first 46 lines for each BAM-file
for f in *.recal.bam; do samtools stats $f -@8|(head -n 46)>>stats.txt; done

# Wolfram script for retrieving the different quality parameters, e.g. for sample 3,
# and plotting the median mean quality (field 39):

# sample=3;
# linesperrecord=46;
# display from =10;
# line=(sample*linesperrecord-(linesperrecord-field));
# outp=Flatten[#]&/@Transpose[{Range[field,linesperrecord,1],d[[line;;sample*linesperrecord]]}];
# Part[outp,All,{1,3,4}]//TableForm

# Median[#[[39]][[3]] & /@ Partition[d, 46]]
# BoxWhiskerChart[#[[39]][[3]] & /@ Partition[d, 46]]
```

Listing 11) Getting general read statistics using samtools flagstats

```
# Assuming that samtools is installed

(for i in *.bam ; do samtools flagstat -@64 $i ; done) >> samtools_flagstat_all.txt
```

Listing 12) General read statistics generated from samtools flagstats

	in total (QC-passed reads	Secondary	Supplementary	Duplicates	mapped (100.00% : N/A)"	Paired in sequencing	Read1	Read2	Properly paired	With itself and mate mapped	Singletons	Mate mapped to a different chr	Mate at diffrent chr (mapQ>=5)
Number of samples	49	49	49	49	49	49	49	49	49	49	49	49	49
Minimum	446309802	195426	0	148013108	446309802	446071685	223555124	222516561	444641035	445756946	314739	617248	578149
25% Percentile	597393975	317610	0	221836226	597393975	596044579	298882752	297161827	592154113	595511408	483341	1303625	1230307
Median	674321211	513998	0	327028137	674321211	674002617	337941391	336061226	668597138	673348974	547319	1938147	1846025
75% Percentile	756860316	736853	0	422159953	756860316	756010517	379382462	376933021	751311213	755349969	616510	2805401	2658981
Maximum	917843320	2476452	0	493128376	917843320	917063488	460244463	456819025	912359382	916093972	969516	4944370	4721920
Range	471533518	2281026	0	345115268	471533518	470991803	236689339	234302464	467718347	470337026	654777	4327122	4143771
Mean	677640886	648444	0	322650844	677640886	676992442	339246232	337746210	673545876	676430663	561779	2136645	2030140
Std. Deviation	119762332	524701	0	100632515	119762332	119723718	60060528	59665400	119071428	119636843	137005	997432	954098
Std. Error of Mean	17108905	74957	0	14376074	17108905	17103388	8580075	8523629	17010204	17090978	19572	142490	136300

Listing 13) Distribution of general read statistics from flagstats

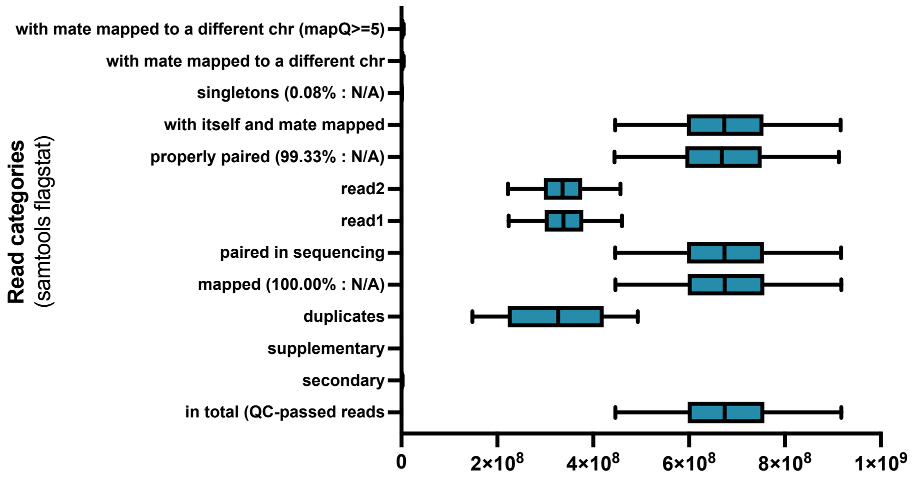

The estimated mean/median unpaired coverage was ~2828x (1872–3850x) when using an exome size of 36.5 Mb[1] (C = LN / G), and a estimated paired end-coverage of ~1413x (935–1923).

Variants were called with HaplotypeCaller and DNA variants were correlated with the RNA set **(Listing 14)**.

1 http://www.twistbioscience.com/products/ngs/fixed-panels/exome2 and http://www.illumina.com/documents/products/technotes/technote_coverage_calculation.pdf

Listing 14) Calling variants through script

```
for f in *.recal.bam; do ./htc.sh ${f/.dedup.recal.bam/}; done
```

Listing 15) Haplotype caller script with preliminary soft variant filtration (flagging)

```
#!/bin/bash

fn=$1'.dedup.recal'

sudo apt update
sudo apt -y install samtools

samtools index $fn'.bam' -@ 8

../gatk-4.3.0.0/gatk --java-options "-Xmx32g" HaplotypeCaller --native-pair-hmm-threads 8 -R ../hg38/re-
sources_broad_hg38_v0_Homo_sapiens_assembly38.fasta -L ../hg38/Twist_Exome_Core_Covered_Targets_hg38.bed -I
$fn'.bam' -O $fn'.vcf'

../gatk-4.3.0.0/gatk IndexFeatureFile -I $fn'.vcf'

../gatk-4.3.0.0/gatk SelectVariants \
-R ../hg38/resources_broad_hg38_v0_Homo_sapiens_assembly38.fasta \
-L ../hg38/Twist_Exome_Core_Covered_Targets_hg38.bed \
-V $fn'.vcf' \
--select-type-to-include SNP \
-O $fn'.SNP.vcf'

../gatk-4.3.0.0/gatk VariantRecalibrator \
-R ../hg38/resources_broad_hg38_v0_Homo_sapiens_assembly38.fasta \
-V $fn'.SNP.vcf' \
--resource:hapmap,known=false,training=true,truth=true,prior=15.0 ../hg38/hapmap_3.3.hg38.vcf.gz \
--resource:omni,known=false,training=true,truth=true,prior=12.0 ../hg38/1000G_omni2.5.hg38.vcf.gz \
--resource:1000G,known=false,training=true,truth=false,prior=10.0 ../hg38/resources_broad_hg38_v0_1000G_pha-
se1.snps.high_confidence.hg38.vcf.gz \
--resource:dbsnp,known=true,training=false,truth=false,prior=2.0 ../hg38/resources_broad_hg38_v0_Homo_sapi-
ens_assembly38.dbsnp138.vcf \
-an QD -an MQ -an MQRankSum -an ReadPosRankSum -an FS -an SOR -an DP \
-mode SNP \
-O $fn'.SNP.recal.vcf' \
--tranches-file $fn'.SNP.output.tranches' \
--rscript-file $fn'.SNP.output.plots.R' \
--dont-run-rscript

../gatk-4.3.0.0/gatk ApplyVQSR \
-R ../hg38/resources_broad_hg38_v0_Homo_sapiens_assembly38.fasta \
-V $fn'.SNP.vcf' \
-O $fn'.SNP.filtered.vcf' \
--truth-sensitivity-filter-level 99.0 \
--tranches-file $fn'.SNP.output.tranches' \
--recal-file $fn'.SNP.recal.vcf' \
-mode SNP

../gatk-4.3.0.0/gatk VariantFiltration \
-R ../hg38/resources_broad_hg38_v0_Homo_sapiens_assembly38.fasta \
-V $fn'.SNP.filtered.vcf' \
-O $fn'.SNP.filtered.dp30.vcf' \
--filter-name "Low_depth30" \
--filter-expression "DP < 30"
```

htc.sh

The intersection of variants between diagnostic, follow-up and control T-cell samples showed a germline concordance (>99.5%, PASS variants) (Listing 16). Also, the DNA-RNA paired variant allele frequency showed a high correlation between diagnosis and follow-up (not shown).

Listing 16) Wolfram script for checking germline concordance

```
Relatedness=Function[fi,
          str="*.dedup.recal.SNP.filtered.dp100.vcf";
          sam=Import[StringReplace[str,"*"->fi[[1]]],"TSV"];
          ctrl=Import[StringReplace[str,"*"->fi[[2]]],"TSV"];
          sa=Part[Select[sam,Length[#]>5&&#[[7]]=="PASS"&],2;;-1,{1,2,4,5}];
          ct=Part[Select[ctrl,Length[#]>5&],2;;-1,{1,2,4,5}];
          Length[Intersection[sa,ct]]/Length[sa]*1.
];

MinMax[{
          Relatedness[{
                    "620_S18_20_0567",
                    "G147-23-0086_twist-exome_HKN3FDSX5"
          }],
          Relatedness[{
                    "620_S4_23_0414",
                    "G147-23-0086_twist-exome_HKN3FDSX5"
          }],
          Relatedness[{
                    "620_S14_23_0448",
                    "G147-23-0086_twist-exome_HKN3FDSX5"
          }],
          Relatedness[{
                    "G147-23-0024_twist-exome_HKN3FDSX5",
                    "G147-23-0086_twist-exome_HKN3FDSX5"
          }],
          Relatedness[{
                    "620_S15_23_0449",
                    "G147-23-0086_twist-exome_HKN3FDSX5"
          }]
}]

{0.99736,0.998074}
```

check_relatedness.nb

Variant annotation was done with a custom developed workflow (Listing 17), where variants with low coverage and common variants are first removed. Secondly, SNPs are removed using the GnomAD database (very comprehensive). ClinVar, COSMIC observations and other annotations were used (see code).

Listing 17) Annotation workflow

```
#!/bin/bash

#rm *.chr*
fn=${1%.vcf}

#s() {

sudo apt-get update && sudo apt-get install bcftools -y

/work/hg38/gatk-4.3.0.0/gatk IndexFeatureFile -I $1

java -jar snpEff/SnpSift.jar annotate -info COMMON 00-common_all.vcf.gz \
$1 | grep -v ";COMMON=1" | grep -v "=chrUn_" | grep -v "=HLA-" | grep -v "VQSRTrancheSNP" > $fn'.uncommon.
vcf'

bcftools view -i 'INFO/DP > 99' $fn'.uncommon.vcf' > $fn'.dbsnp.vcf'

/work/hg38/gatk-4.3.0.0/gatk IndexFeatureFile -I $fn'.dbsnp.vcf'


counter=1
while [ $counter -le 22 ]
do
echo $counter

/work/hg38/gatk-4.3.0.0/gatk SelectVariants \
     -R Homo_sapiens_assembly38.fasta \
     -V $fn'.dbsnp.vcf' \
     -L 'chr'$counter \
     -O $fn'.chr'$counter'.vcf' &


counter=$(($counter+1))

done

/work/hg38/gatk-4.3.0.0/gatk SelectVariants \
     -R Homo_sapiens_assembly38.fasta \
```

```
     -V $fn'.dbsnp.vcf' \
     -L 'chrX' \
     -O $fn'.chrX.vcf' &

/work/hg38/gatk-4.3.0.0/gatk SelectVariants \
     -R Homo_sapiens_assembly38.fasta \
     -V $fn'.dbsnp.vcf' \
     -L 'chrY' \
     -O $fn'.chrY.vcf' &


wait

counter=1
while [ $counter -le 22 ]
do
        echo $counter

        java -jar snpEff/SnpSift.jar annotate -name gnomAD. \
        -info AF 'v4/gnomad.exomes.v4.0.sites.chr'$counter'.vcf.bgz' \
        $fn'.chr'$counter'.vcf' > $fn'.chr'$counter'.gnomad.vcf' &

        counter=$(($counter+1))

done

java -jar snpEff/SnpSift.jar annotate -name gnomAD. \
-info AF 'v4/gnomad.exomes.v4.0.sites.chrX.vcf.bgz' \
$fn'.chrX.vcf' > $fn'.chrX.gnomad.vcf' &

java -jar snpEff/SnpSift.jar annotate -name gnomAD. \
-info AF 'v4/gnomad.exomes.v4.0.sites.chrY.vcf.bgz' \
$fn'.chrY.vcf' > $fn'.chrY.gnomad.vcf' &

wait

/work/hg38/gatk-4.3.0.0/gatk GatherVcfs \
-I $fn'.chr1.gnomad.vcf' \
-I $fn'.chr2.gnomad.vcf' \
-I $fn'.chr3.gnomad.vcf' \
-I $fn'.chr4.gnomad.vcf' \
-I $fn'.chr5.gnomad.vcf' \
-I $fn'.chr6.gnomad.vcf' \
-I $fn'.chr7.gnomad.vcf' \
-I $fn'.chr8.gnomad.vcf' \
-I $fn'.chr9.gnomad.vcf' \
-I $fn'.chr10.gnomad.vcf' \
-I $fn'.chr11.gnomad.vcf' \
-I $fn'.chr12.gnomad.vcf' \
-I $fn'.chr13.gnomad.vcf' \
-I $fn'.chr14.gnomad.vcf' \
-I $fn'.chr15.gnomad.vcf' \
-I $fn'.chr16.gnomad.vcf' \
-I $fn'.chr17.gnomad.vcf' \
-I $fn'.chr18.gnomad.vcf' \
-I $fn'.chr19.gnomad.vcf' \
-I $fn'.chr20.gnomad.vcf' \
-I $fn'.chr21.gnomad.vcf' \
-I $fn'.chr22.gnomad.vcf' \
-I $fn'.chrX.gnomad.vcf' \
-I $fn'.chrY.gnomad.vcf' \
-O $fn'.gnomad.vcf'


bcftools view -e 'INFO/gnomAD.AF >= 0.01' $fn'.gnomad.vcf' > $fn'.gnomad.nonSNP.vcf'
java -jar snpEff/SnpSift.jar annotate -info CLNSIG /work/hg38/clinvar_20221211.vcf.gz $fn'.gnomad.nonSNP.vcf'
> $fn'.gnomad.nonSNP.clinvar.vcf'

java -jar snpEff/snpEff.jar GRCh38.p13 $fn'.gnomad.nonSNP.clinvar.vcf' > $fn'.gnomad.nonSNP.clinvar.snpeff.vcf'

java -jar snpEff/SnpSift.jar annotate -info PON /work/hg38/somatic-hg38-1000g_pon.hg38.mod.vcf.gz $fn'.gno-
mad.nonSNP.clinvar.snpeff.vcf' | grep -v "PON=1" > $fn'.gnomad.nonSNP.clinvar.snpeff.nonpon.vcf'

java -jar snpEff/SnpSift.jar annotate -info CNT /work/hg38/CosmicCodingMuts.vcf.gz $fn'.gnomad.nonSNP.clin-
var.snpeff.nonpon.vcf' > $fn'.gnomad.nonSNP.clinvar.snpeff.nonpon.cosmic.vcf'

java -jar snpEff/SnpSift.jar dbnsfp -v -db /work/hg38/dbNSFP4.1a.txt.gz $fn'.gnomad.nonSNP.clinvar.snpeff.non-
```

```
pon.cosmic.vcf' > $fn'.gnomad.nonSNP.clinvar.snpeff.nonpon.cosmic.dbnsfp.vcf'
#}
/work/hg38/gatk-4.3.0.0/gatk IndexFeatureFile -I $fn'.gnomad.nonSNP.clinvar.snpeff.nonpon.cosmic.dbnsfp.vcf'

/work/hg38/gatk-4.3.0.0/gatk VariantsToTable --show-filtered -V $fn'.gnomad.nonSNP.clinvar.snpeff.nonpon.cos-
mic.dbnsfp.vcf' -F CHROM -F POS -F REF -F ALT -F ID -F TYPE -F FILTER -F CNT -GF AD -GF AF -F ANN -O $fn'.
gnomad.nonSNP.clinvar.snpeff.nonpon.cosmic.dbnsfp.table'

grep -f /work/hg38/QuickGO-annotations_B-cell_symbols.txt $fn'.gnomad.nonSNP.clinvar.snpeff.nonpon.cosmic.
dbnsfp.table' > $fn'.annotated.table.B-cellactivation.tsv'

/work/hg38/gatk-4.3.0.0/gatk VariantsToTable --show-filtered -V $fn'.gnomad.nonSNP.clinvar.snpeff.nonpon.cos-
mic.dbnsfp.vcf' -F CHROM -F POS -F REF -F ALT -F ID -F TYPE -F FILTER -F CNT -GF AD -GF AF -F ANN -F dbNS-
FP_LRT_pred -F dbNSFP_MetaSVM_pred -F dbNSFP_MutationAssessor_pred -F dbNSFP_MutationTaster_pred -F dbNSFP_
PROVEAN_pred -F dbNSFP_Polyphen2_HDIV_pred -F dbNSFP_Polyphen2_HVAR_pred -O $fn'.out.tsv'


# Note: generate GO-symbol list by cat QuickGO-annotations_B-cell.tsv | cut -f3 | uniq | awk '{print "|" $1
"|"};' > QuickGO-annotations_B-cell_symbols.txt

mkdir -p tmp
mv $fn*'.vcf' tmp/.
mv $fn*'.vcf.idx' tmp/.
mv $fn*'.tsv' tmp/.
mv $fn*'.table' tmp/.
mv 'tmp/'$fn'.gnomad.nonSNP.clinvar.snpeff.nonpon.cosmic.dbnsfp.vcf' .
mv 'tmp/'$fn'.out.tsv' .
mv 'tmp/'$1 .
```

annotatesomatic_050224.sh

Variants were processed and paired using Wolfram script in Mathematica and exported to Excel for further analyses (Listing 18).

Listing 18) Wolfram script for importing and formatting variants (VCF) and exporting for Excel

```
<<JLink`;
InstallJava[];
ReinstallJava[JVMArguments->"-Xmx4024m"];
SetDirectory[NotebookDirectory[]];


gwm=Import["Genes_with_mutation_2023.txt","TSV"];
gwmlist=Select[Reverse[SortBy[Flatten[{#,#[[2]]/#[[3]]*1.}]&/@Select[gwm[[2;;-1]],Length[StringSplit[#[[1]],"_"]]==1&],La
st]],#[[-1]]>0.01&&#[[2]]>1&];
gwmlist=Part[gwmlist,All,1];

fi=FileNames["*out.tsv"]
sym=Import["QuickGO-annotations_B-cell_symbols.txt","TSV"];
sym=StringReplace[Flatten[sym[[2;;-1]]],"|"->""];

importNxl=Function[f,
   z=Import[f,"Text"];
   z=StringReplace[z,","->";"];
   z=StringSplit[#,"\t"]&/@StringSplit[z,"\n"];
   z=Select[z,StringContainsQ[#[[7]],"multiallelic"]==False&&StringContainsQ[#[[7]],"panel_of_normals"]==False&];
   zo=Flatten[{symbol=StringSplit[#[[9]],"|"][[4]],If[MemberQ[sym,symbol] || MemberQ[gwmlist,symbol],1,0],StringSplit[#[[9]],"|"][[3]],
   StringSplit[#[[9]],"|"][[2]],#}]&/@z[[2;;-1]];
   Export[f<>".format2.tsv",Join[{Flatten[{{"Symbol","B-cell activation","Effect","Type"},z[[1]]}]},zo],"Table"];
];

GetOccurrences=Function[in,
   Flatten[Select[MutList,#[[1;;4]]==in&]][[5]]
];

p = {"620", "623", "664", "670", "692", "705", "741", "748", "875",
   "911", "923"};

patient["620"] = {
   "620_S4_23_0414",
   "620_S14_23_0448",
   "620_S15_23_0449",
   "620_S18_20_0567",
   "G147-23-0024_twist-exome_HKN3FDSX5"
   };

patient["623"] =
  {
   "623_S1_23_0384",
   "623_S5_23_0421",
   "623_S12_23_0446",
   "623_S19_20_0569",
   "G147-23-0017_twist-exome_HKN3FDSX5"
   };
```

```
patient["664"] =
  {
   "664_S6_23_0422",
   "664_S13_23_0447",
   "664_S17_20_0566",
   "G147-23-0025_twist-exome_HKN3FDSX5"
   };

patient["670"] =
  {
   "670_S22_22_0077",
   "G147-23-0032_twist-exome_HKN3FDSX5"
   };

patient["692"] =
  {
   "692_S2_23_0412",
   "692_S10_23_0444",
   "692_S11_23_0445",
   "G147-23-0035_twist-exome_HKN3FDSX5"
   };

patient["705"] =
  {
   "705_S3_23_0413",
   "705_S9_23_0443",
   "705_S20_21_0336",
   "G147-23-0020_twist-exome_HKN3FDSX5"
   };

patient["741"] =
  {
   "741_S21_22_0076",
   "G147-23-0033_twist-exome_HKN3FDSX5"
   };

patient["748"] =
  {
   "748_S23_22_0078",
   "G147-23-0042_twist-exome_HKN3FDSX5"
   };

patient["875"] =
  {
   "875_S16_23_0455",
   "875_S25_23_0442",
   "G147-23-0034_twist-exome_HKN3FDSX5"
   };

patient["911"] =
  {
   "911_S7_23_0428",
   "911_S24_23_0441",
   "G147-23-0018_twist-exome_HKN3FDSX5"
   };

patient["923"] =
  {
   "923_S8_23_0430",
   "923_S26_23_0457",
   "G147-23-0015_twist-exome_HKN3FDSX5",
   "G147-23-0043_twist-exome_HKN3FDSX5"
   };

Flatten[patient[#] & /@ p];


(* FORMAT ALL FILES *)

ext="_somatic_filtered.gnomad.nonSNP.clinvar.snpeff.nonpon.cosmic.dbnsfp.out.tsv";
importNxl[#<>ext]&/@patient[#]&/@p[[1;;-1]];


VAFthreshold=Function[in,
   Select[in, MemberQ[highVAFs,#[[5;;8]]]&]
]

ExportSomatics=Function[pid,
   ext="_somatic_filtered.gnomad.nonSNP.clinvar.snpeff.nonpon.cosmic.dbnsfp.out.tsv.format2.tsv";
   patientdata=Import[#<>ext,"TSV"]&/@patient[pid];
   header=Join[#[[1]],{"Tumor REF reads","Tumor ALT reads","CTRL REF reads","CTRL ALT reads",
   "Present in no. of patient samples"}]&/@patientdata;

   MutList=Flatten[#]&/@Tally[Flatten[Part[#,2;;-1,{5,6,7,8}]&/@patientdata,1]];
   out=Flatten[{#,StringSplit[#[[-4]],";"],StringSplit[#[[-2]],";"],GetOccurrences[#[[5;;8]]]}]&/@#[[2;;-1]]&/@patientdata;

   highVAFs=DeleteDuplicates[Part[Select[Flatten[Part[out,All,1;;-1,{5,6,7,8,-8}],1],#[[-1]]>0.1&],All,1;;-2]];
   out=VAFthreshold[#]&/@out;

   out=Prepend[#[[2]],#[[1]]]&/@Transpose[{header,out}];
   dataout=Flatten[{#[[1]]->#[[2]]}&/@Transpose[{patient[pid],Reverse[SortBy[#,#[[-8]]&]]&/@out}],1];

   Export[pid<>"_combined_somaticvariants_Jan2024.xlsx",dataout,"XLSX"]

];


(* EXPORT AS EXCEL *)

ExportSomatics[#]&/@p
```

After sample pairing, the variants were paired with variants from RNA-sequencing workflow (**Listing 19**). See separate documentation for this specific workflow.

Listing 19) Importing RNA variants and expression profiles for correlation with exome variants

```
SetDirectory[NotebookDirectory[]];

FormatSampleID=Function[in,
   str=StringSplit[in,"_"];
   str[[1]]<>"_"<>str[[2]]
];

FormatReadNum=Function[in,
   str=StringSplit[in,{":",","}];
   If[Length[str]>1,
         ref=ToExpression[str[[2]]];
         alt=ToExpression[str[[3]]];
         total=ref+alt;
         r="DP:"<>ToString[total]<>", AF:"<>ToString[Round[alt/total,0.01]];
         ,
         r="NA";
   ];
   r
];

SelectInline=Function[{s,exp},
   Select[s,exp]
];

path="HPF/";
RNAfiles=FileNames[path<>"CLL_RNA/CLLrna_vcfs/*"];
samples=Select[Import[path<>"CLL_RNA/RNAsamples.xlsx"][[1]],#[[1]]!=""&][[2;;-1]];

Unset[s]
(s[#[[1]]]=ToString[Round[#[[3]]]])&/@samples;
rna={Transpose[{ConstantArray[StringReplace[#,{"path<>"CLL_RNA/CLLrna_vcfs/"->"",".filtered.dp100.vcf"->""}],
Length[Import[#,"TSV"]]],Import[#,"TSV"]}]}&/@RNAfiles[[1;;-1]];

rna=Flatten[#]&/@#&/@#&/@rna;
rna=Flatten[rna,2];
rna=Select[rna,Length[#]>2&];


RNAexpressionfiles=FileNames["path<>"CLL_RNA/expressionprofiles/*.symbolcounts.tsv"];
FormatSampleID[StringSplit[#,{"path<>"CLL_RNA/expressionprofiles/","_trim.symbolcounts.tsv"}][[1]]]&/@RNAexpressionfiles;

ImportExpressions=Function[f,
   exps=Import[f,"TSV"];
   sID=FormatSampleID[StringSplit[f,{"path<>"CLL_RNA/expressionprofiles/","_trim.symbolcounts.tsv"}][[1]]];
   pID=s[sID];

   Part[Flatten[#]&/@Transpose[{ConstantArray[pID,Length[exps]],ConstantArray[sID,Length[exps]],exps}],2;;-1,{1,2,4,-1}]
];

GlobalExp=ImportExpressions[#]&/@RNAexpressionfiles;
GlobalExp=Flatten[GlobalExp,1];


PairNexport=Function[pt,

   ptrna=Quiet[Flatten[{#,s[FormatSampleID[#[[1]]]],"Pt:"<>s[FormatSampleID[#[[1]]]]<>" ("<>#[[1]]<>"), "<>FormatReadNum[#[[-1]]]}]&/@rna];
   ptrna=Select[ptrna,#[[-2]]==pt&];

   v=Import["path<>"WES_CLL_followup_pilot24/Filtered_pt"<>pt<>"_combined_somaticvariants_Jan2024.xlsx"];
   sheetnames=Import[path<>"WES_CLL_followup_pilot24/Filtered_pt"<>pt<>"_combined_somaticvariants_Jan2024.xlsx","Sheets"];
   sheetnames=StringReplace[#,{"_twist-exome_HKN3FD"->"","-"->"_"}]&/@sheetnames;


   ptGE=Select[GlobalExp,#[[1]]==pt&];
   ptGE=Flatten[{index=#[[2]];Select[sheetnames,StringContainsQ[#,index]&],#[[-2;;-1]]}]&/@ptGE;


   xx=Flatten[#]&/@Transpose[{ConstantArray[#[[1]],Length[#[[2]]]],#[[2]]}]&/@Transpose[{sheetnames,v}];

   rnapair=Flatten[{#[[2;;-1]],id=#[[1]];gene=#[[2]];SelectInline[ptGE,#[[1]]==id&&#[[2]]==gene&][[-1]],a=#[[{6,7,8,9}]];b=#[[1]];Part[SelectIn-
   line[ptrna,#[[{2,3,5,6}]]==a&&StringContainsQ[b,StringSplit[#[[1]],"_S"][[1]]]&],All,-1]}]&/@#&/@xx[[1;;-1]];


   Export[pt<>"_MutectNRNA3.xlsx", Flatten[{#[[1]]->#[[2]]}&/@Transpose[{sheetnames,rnapair}],1],"XLSX"]

];
PairNexport["620"]
PairNexport["923"]
PairNexport["911"]
PairNexport["875"]
PairNexport["748"]
PairNexport["741"]
PairNexport["705"]
PairNexport["692"]
PairNexport["670"]
PairNexport["664"]
PairNexport["623"]
```

Finally, the copy-number assessment was performed using BEDtools multicov-generated coverage files (Listing 20) and variant allele frequencies (Listing 21). An example of these plots is provided in (Listing 22). The accompanying excel output is shown in (Listing 23), showing nearly 100% clonal burden of trisomy 12.

Listing 20) Getting paired coverage profiles

```
#!/bin/bash

tumor=$1
control=$2
bedtools multicov -bams $tumor'_twist-exome_HKN3FDSX5.dedup.recal.bam' $control'_twist-exome_HKN3FDSX5.de-
dup.recal.bam' -bed Twist_Exome_Core_Covered_Targets_hg38.bed > 'BED/G147-23-'$tumor'_twist-exome_HKN3FDSX5.
dedup.recal.paired.bed'
```

Listing 21) Wolfram script for creating VAF-based copy-number alteration plots and excel output files

```
Needs["HypothesisTesting`"];
SetDirectory[NotebookDirectory[]];


(*Apply Yates' continuity correction*)
Chi2Test2=Function[{e,i,o,u},
   {a,b,c,d}={e,i,o,u}+1/2.;
   chi2=(Total[{a,b,c,d}]*(b*c-a*d)^2)/((a+b)*(a+c)*(b+d)*(c+d));
   chi2=SetPrecision[chi2,20];
   p=ChiSquarePValue[chi2,1][[2]];
   If[chi2>1,p,1-p]
];


ImportNPair2=Function[{tumor,control},

   tvcf=Import[tumor,"TSV"];
   cvcf=Import[control,"TSV"];

   vcf=Join[tvcf,cvcf];
   vcf=Select[vcf,Length[#]>1&&#[[7]]=="PASS"&];
   vcf=Flatten[{#[[1;;4]],ToExpression[StringSplit[#[[-1]],{":",","}][[2;;3]]]}]&/@vcf;

   vcf=Part[vcf,All,{1,2,3,4,-2,-1}];

   vcf=Select[GatherBy[vcf,#[[1;;4]]&],Length[#]==2&];

   vcf=Flatten[{#[[1]],#[[2]][[-2;;-1]]}]&/@vcf;
   vcf=Flatten[{#,Apply[Chi2Test2,#[[-4;;-1]]]}]&/@vcf;
   vcf=Flatten[{#,#[[-4]]/Total[#[[-5;;-4]]]*1.,#[[-2]]/Total[#[[-3;;-1]]]*1.}]&/@vcf;

];


getCNA=Function[file,
   PartionSize=20; (*20*);
   Gaussianthreshold=10; (*9*)
   bed=Import[file,"TSV"];
   b=Select[bed,NumberQ[#[[-2]]]&&NumberQ[#[[-1]]]&&Total[#[[-2;;-1]]]>=100&];
   n=Flatten[{#,l=(#[[3]]-#[[2]]),#[[4]]/l*1.,#[[5]]/l*1.}]&/@b;

   position=Part[n,All,1;;3];
   position=Flatten[{#[[1]][[1;;2]],#[[-1]][[-1]]}]&/@Partition[position,PartionSize];

   t=Part[n,All,-2];
   t=t/Median[t]*1.;

   c=Part[n,All,-1];
   c=c/Median[c]*1.;

   a=Transpose[{t,c}];
   a=If[#[[1]]==0||#[[2]]==0,0,#[[1]]/#[[2]]*1.]&/@a;
   a=Median[#]&/@Partition[a,PartionSize];

   U=Transpose[#]&/@Partition[Transpose[{GaussianFilter[t,Gaussianthreshold],GaussianFilter[c,Gaussianthreshold]}],PartionSize];
   Utest=MannWhitneyTest[#]&/@U;

];


CreateVAFplot=Function[inputfiles,

   ImportNPair2[inputfiles[[1]],inputfiles[[2]]];
   getCNA[inputfiles[[3]]];

   GetU=Function[pos,
   Select[x[pos[[1]]],#[[2]]<pos[[2]]<#[[3]]&]
   ];


   ua=Flatten[#]&/@Transpose[{position,a,Utest}];

   {x[#[[1]][[1]]]=#}&/@GatherBy[ua,First];

   u=Flatten[{#,GetU[#[[1;;3]]]}]&/@vcf[[1;;-1]];
```

```
   Clear[x];

   pval=0.05;
   filterthreshold=20; (

   vcfchrlist=Part[vcf,All,1];
   chrpos=#[[1]]&/@GatherBy[Transpose[{Range[Length[vcfchrlist]],vcfchrlist}],Last];
   lines=Part[chrpos,All,-2];

   both=If[#[[1]]<pval&&#[[2]]<pval,Mean[#],1]&/@Transpose[{MedianFilter[Part[u,All,9],filterthreshold],MedianFilter[Part[u,All,-1],filterthres-
   hold]}];

   p1=ListPlot[{Part[vcf,All,-1],Part[vcf,All,-2],Callout[{#[[1]],0.01},StringReplace[#[[2]],"chr"->""],Above]&/@chrpos}];
   p2=ListLinePlot[both,Filling->Axis,FillingStyle->Directive[Red,Opacity[0.05]],PlotStyle->Directive[Red,Opacity[0.1]],GridLines->{lines}];
   plot=Rasterize[Show[p2,p1,PlotLabel->Style[Framed[inputfiles[[1]]],16,Black,Background->Lighter[LightGray]],AxesLabel->{"Variants","VAF"},La-
   belStyle->Directive[Gray,14,Bold],PlotRange->{0,1}],ImageSize->{850,550}];
   Export[inputfiles[[1]]<>".pariedCNAplot.jpg",plot,"JPEG"];

   header={"Chromosome","Position","Reference","Variant","Reference depth","Variant depth", "Reference depth (control)", "Variant depth (con-
   trol)","Pval Chi^2","VAF", "VAF (control)","Target chr", "Target start", "Interval end","Relative copy number","Pval U test", "Smoothened
   Pval"};

   Export[inputfiles[[1]]<>".pairedCNAs.xlsx",Prepend[Select[Flatten[#]&/@Transpose[{u,both}],#[[-1]]<pval&&#[[9]]<pval&&Length[#]==17&],hea-
   der],"XLSX"];


   plot

];


ImportNPair=Function[{tumor,control},

   tvcf=Import[tumor,"TSV"];
   cvcf=Import[control,"TSV"];

   vcf=Join[tvcf,cvcf];
   vcf=Select[vcf,Length[#]>1&&#[[7]]=="PASS"&];
   vcf=Part[vcf,All,{1,2,4,5}];
   total=Length[vcf];
   verified =Tally[vcf];
   Round[2*Length[Select[verified,#[[2]]==2&]]/total,0.0001]

];

pairinglist = \
{
          {
                    "G147-23-0020_twist-exome_HKN3FDSX5.dedup.recal.SNP.filtered.dp100.vcf",
                    "G147-23-0074_twist-exome_HKN3FDSX5.dedup.recal.SNP.filtered.dp100.vcf",
                    "G147-23-0020_twist-exome_HKN3FDSX5.dedup.recal.paired.bed"
          },{
                    "G147-23-0015_twist-exome_HKN3FDSX5.dedup.recal.SNP.filtered.dp100.vcf",
                    "G147-23-0075_twist-exome_HKN3FDSX5.dedup.recal.SNP.filtered.dp100.vcf",
                    "G147-23-0015_twist-exome_HKN3FDSX5.dedup.recal.paired.bed"
          },
... };


ImportNPair[#[[1]],#[[2]]]&/@Part[pairinglist,All,1;;-1]
```

Listing 22) Variant allele-frequency copy-number alteration plots example showing trisomy 12

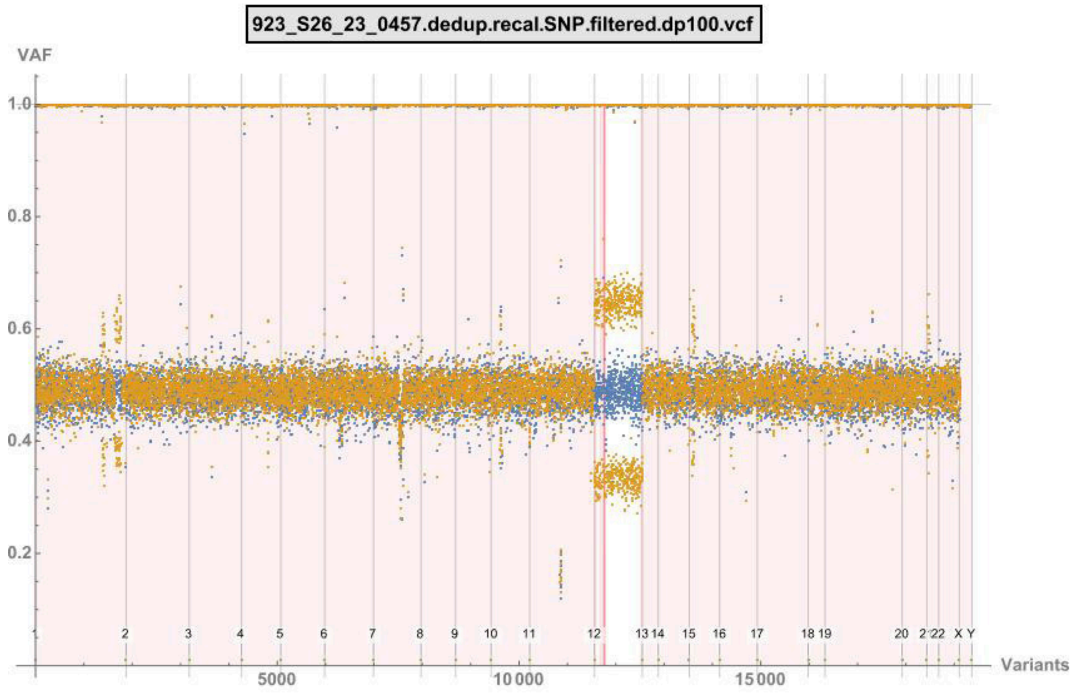

Listing 23) Chromosome 12 trisomy example output (truncated and numbers rounded)

Chr	Position	Variant	Variant depth	Variant depth (control)	Pval Chi^2	VAF	VAF (control)	Target start	Interval end	Relative copy number	P-value (U test)	Smoothened Pval
chr12	223196	C	903	638	$8.55 \cdot 10^{-17}$	0.65	0.49	196123	237290	1.64	$6.80 \cdot 10^{-8}$	$1.75 \cdot 10^{-4}$
chr12	322420	T	760	539	$2.52 \cdot 10^{-23}$	0.65	0.45	237925	331938	1.41	$6.80 \cdot 10^{-8}$	$7.41 \cdot 10^{-5}$
chr12	411781	A	852	712	$1.47 \cdot 10^{-13}$	0.63	0.49	333486	438554	1.46	$6.80 \cdot 10^{-8}$	$2.89 \cdot 10^{-5}$
chr12	548238	G	416	722	$1.04 \cdot 10^{-20}$	0.32	0.50	440616	558088	1.59	$6.80 \cdot 10^{-8}$	$7.41 \cdot 10^{-5}$
chr12	552490	A	470	704	$2.43 \cdot 10^{-22}$	0.32	0.50	440616	558088	1.59	$6.80 \cdot 10^{-8}$	$2.89 \cdot 10^{-5}$
chr12	553282	T	1335	1575	$5.10 \cdot 10^{-06}$	0.98	1.00	440616	558088	1.59	$6.80 \cdot 10^{-8}$	$2.89 \cdot 10^{-5}$
chr12	553672	T	331	527	$2.76 \cdot 10^{-09}$	0.33	0.45	440616	558088	1.59	$6.80 \cdot 10^{-8}$	$2.89 \cdot 10^{-5}$
chr12	753986	G	212	304	$1.17 \cdot 10^{-12}$	0.31	0.51	558507	880031	1.48	$6.80 \cdot 10^{-8}$	$2.89 \cdot 10^{-5}$
chr12	830136	A	480	813	$3.75 \cdot 10^{-24}$	0.32	0.50	558507	880031	1.48	$6.80 \cdot 10^{-8}$	$2.58 \cdot 10^{-6}$
chr12	859323	T	455	803	$4.10 \cdot 10^{-23}$	0.33	0.51	558507	880031	1.48	$6.80 \cdot 10^{-8}$	$2.38 \cdot 10^{-6}$
chr12	878316	G	604	809	$7.50 \cdot 10^{-16}$	0.33	0.46	558507	880031	1.48	$6.80 \cdot 10^{-8}$	$1.39 \cdot 10^{-6}$
chr12	881746	A	906	759	$1.29 \cdot 10^{-19}$	0.66	0.49	880720	914532	1.48	$6.80 \cdot 10^{-8}$	$7.52 \cdot 10^{-7}$
chr12	884848	C	421	726	$3.52 \cdot 10^{-19}$	0.31	0.47	880720	914532	1.48	$6.80 \cdot 10^{-8}$	$7.52 \cdot 10^{-7}$
chr12	889199	G	1019	754	$8.40 \cdot 10^{-24}$	0.66	0.48	880720	914532	1.48	$6.80 \cdot 10^{-8}$	$3.89 \cdot 10^{-7}$
chr12	1371855	A	453	719	$5.19 \cdot 10^{-23}$	0.32	0.50	1236768	1784073	1.51	$6.80 \cdot 10^{-8}$	$3.89 \cdot 10^{-7}$
chr12	1781033	G	431	864	$2.23 \cdot 10^{-39}$	0.28	0.51	1236768	1784073	1.51	$6.80 \cdot 10^{-8}$	$3.89 \cdot 10^{-7}$
chr12	1783911	C	291	380	$3.58 \cdot 10^{-10}$	0.31	0.46	1236768	1784073	1.51	$6.80 \cdot 10^{-8}$	$3.89 \cdot 10^{-7}$
chr12	1784004	C	395	576	$6.82 \cdot 10^{-14}$	0.35	0.50	1236768	1784073	1.51	$6.80 \cdot 10^{-8}$	$1.73 \cdot 10^{-7}$
chr12	1840773	C	696	428	$1.67 \cdot 10^{-13}$	0.61	0.45	1785943	1854044	1.74	$6.80 \cdot 10^{-8}$	$3.89 \cdot 10^{-7}$
chr12	1874616	C	941	695	$1.22 \cdot 10^{-24}$	0.63	0.45	1856011	1913139	1.58	$6.80 \cdot 10^{-8}$	$1.73 \cdot 10^{-7}$
chr12	1879001	T	851	575	$1.47 \cdot 10^{-10}$	0.62	0.49	1856011	1913139	1.58	$6.80 \cdot 10^{-8}$	$1.73 \cdot 10^{-7}$
chr12	1879852	T	668	528	$2.79 \cdot 10^{-07}$	0.63	0.52	1856011	1913139	1.58	$6.80 \cdot 10^{-8}$	$1.15 \cdot 10^{-7}$

Veyhe et al. 2024. Supplement. Part III

RNA SEQUENCING OF CLL FOLLOW-UP SAMPLES

Number of samples sequenced	42 (32 from sorted B cells, 10 PBMC)
Sequencing details	Sequenced on NovaSeq 6000, S2 flow cell XP, Lane 1 (Lane 2 was 10X scRNA), Amplicon pool conc. nM: 4,36, concentration (nM) for Novaseq: 0,5, Pool µl: 2,52, EB-buffer used 19,48 µl. Novaseq config: NaOH: 10 µl 2N NaOH + 90 µl H2O, PhiX: 1 µl + 3 µl EB, Tris: 40 µl Tris + 60 µl H2O
Compiled by	Marcus Høy Hansen, marcus.hoy.hansen@rsyd.dk

Introduction

Bulk transcriptome sequencing was performed on RNA from the same samples as those used for the DNA sequencing previously described (see Supplement, part II). RNA was extracted, and its concentration and RNA integrity number (RIN) were assessed using a Bioanalyzer (Agilent). The median retrievable RIN value for the B cell samples was 8 (range 2.5 to 9.4), with a median RNA amount of 71.6 ng (3.4–1863 ng). The median RIN value for the PBMC samples was 2.4 (2.2–4.7), with a median RNA amount of 7.8 ng (2–29.6 ng).

RNA libraries were prepared using the Illumina Stranded mRNA Prep Kit (Illumina), adhering to the manufacturer's guidelines and ensuring comprehensive coding transcriptome coverage. Sequencing was conducted on the NovaSeq 6000 platform (Illumina), utilizing an S2 flow cell XP (Illumina).

Bioinformatics

The bioinformatics pipeline partly adopted the Broad Institute GATK Best Practices Workflow, which focuses on RNA-seq short variant discovery (**Listing 1**). No duplicates were marked for variant discovery. Because a high-resolution set was already defined from deep whole-exome sequencing, only flagging/soft filtering and minimal variant annotation were needed for comparison.

Listing 1) The workflow was adapted from Broad Institute's Best Practices workflow on RNAseq short variant discovery

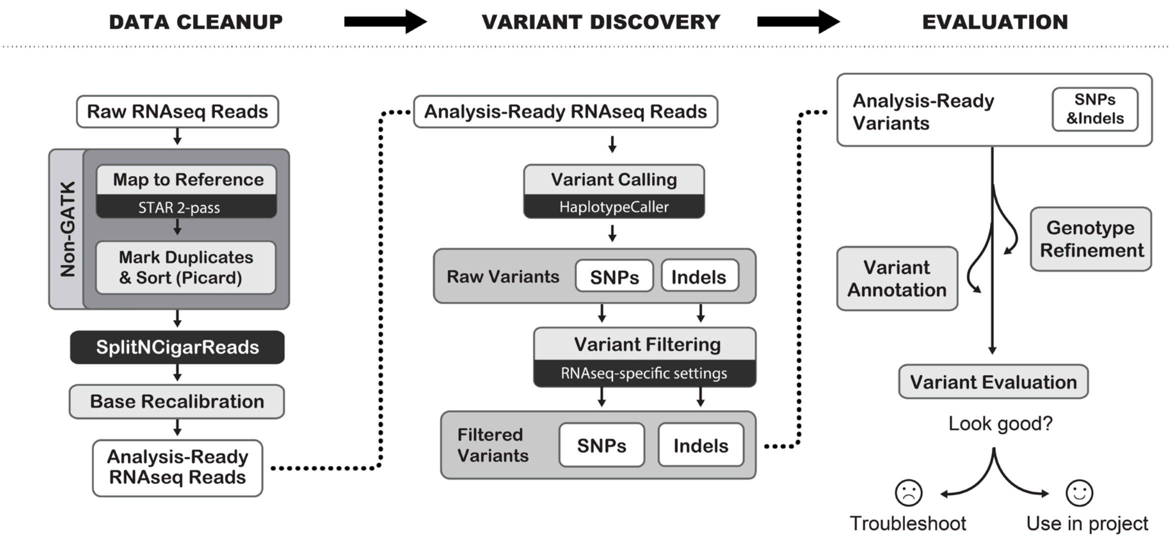

source: gatk.broadinstitute.org, RNAseq short variant discovery (SNPs + Indels), accessed January 2024

Due to 3’ adapter sequences and 5’ “jitter” **(Listing 2)**, presumably from random hexa-/nonamer priming[1], the FASTQ files were trimmed using multithreaded cutadapt (32 vCPU machine). Installation was performed using conda **(Listing 3)** and run with 31 threads **(Listing 4)** on Ucloud.

Listing 2) 3’ Nextera Transposase sequence contamination and 3’ bias from random priming

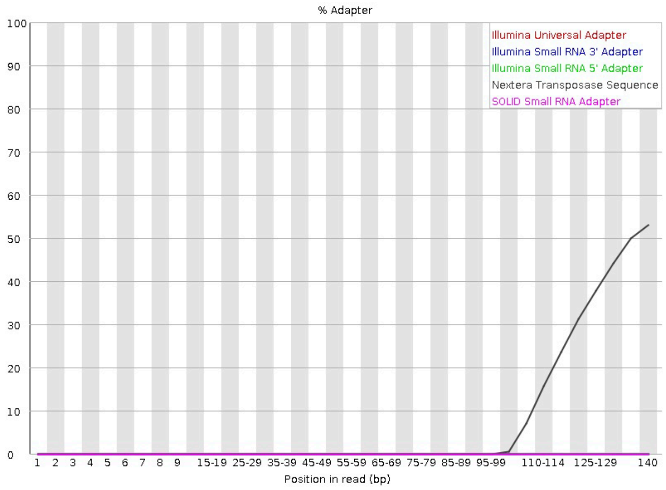

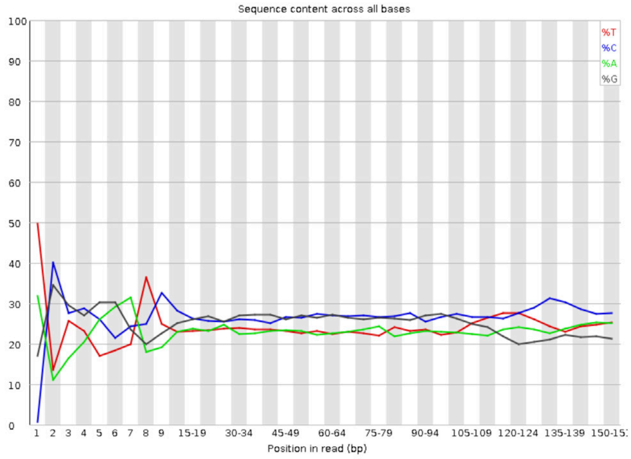

Listing 3) Installing cutadapt for trimming

```
# Get Miniconda3
wget https://repo.anaconda.com/miniconda/Miniconda3-latest-Linux-x86_64.sh

# Install Miniconda3 (parameters -u -g may be used for unsupervised installation)
bash Miniconda3-latest-Linux-x86_64.sh

# Initialize and reinitialize terminal (or restart terminal)
/ucloud/miniconda3/bin/conda init
source ~/.profile

# Install cutadapth with a working version of python
 conda create -n cutadapt -c conda-forge -c bioconda cutadapt python=3.9

# Activate environment
conda activate cutadapt
```

Listing 4) Using cutadapt for trimming

```
#!/bin/bash

# Read 1 from paired-end sequencing (remember to activate conda environment)
cutadapt -u 12 -u -50 -o $1’_trim_R1_001.fastq.gz’ $1’_R1_001.fastq.gz’ --cores=31

# Read 2 from paired-end sequencing
cutadapt -u 12 -u -50 -o $1’_trim_R2_001.fastq.gz’ $1’_R2_001.fastq.gz’ --cores=31
```

file: cutadapt.sh

Trimming script was invoked for all FASTQ files in folders with sample ID as parameter **(Listing 5)**.

Listing 5) Trim all files (in interface terminal)

```
# Remember to make cutadapt executable using chmod u+x cutadapt.sh
ext=’_R1_001.fastq.gz’
for f in *$ext; do cutadapt.sh ${f/$ext/}; done
```

1 http://hbctraining.github.io/Intro-to-rnaseq-hpc-salmon/lessons/qc_fastqc_assessment.html

No deduplication were performed because of (1) the need for deduplication is not clearly an advantage for RNA sequencing[2] (see foot note), (2) few samples were of low DNA-input and total reads, and thus potentially detrimental for the resolution.

STAR2 (version STAR-2.7.10b) was run from the downloaded script (Listing 6).

Listing 6) Installing STAR

```
# Get latest STAR source from releases
wget https://github.com/alexdobin/STAR/archive/2.7.10b.tar.gz
tar -xzf 2.7.10b.tar.gz
cd STAR-2.7.10b

# Alternatively, get STAR source using git
# git clone https://github.com/alexdobin/STAR.git

# Compile
cd source
make STAR
```

source: http://github.com/alexdobin/STAR

STAR2 ran using 31 threads for the alignment of the trimmed FASTQ files, but can be extended for increased speed (64 vCPUs). The reference genome is not compatible with BWA and has to be built together with the annotation file (Listing 7). In this case, the STAR38 (GRCh38 compatible) reference genome has been built for a previous project. STAR2 was called through a created bash script (Listing 8).

Listing 7) Excerpt from STAR manual on genomeGenerate

The basic options to generate genome indices are as follows:

```
--runThreadN NumberOfThreads
--runMode genomeGenerate
--genomeDir /path/to/genomeDir
--genomeFastaFiles /path/to/genome/fasta1 /path/to/genome/fasta2 ...
--sjdbGTFfile /path/to/annotations.gtf
--sjdbOverhang ReadLength-1
```

source: github.com/alexdobin/STAR/blob/master/doc/STARmanual.pdf

Listing 8) STAR script

```
#!/bin/bash

# Invoked with ./runstar.sh [sampleID]'_trim'

mkdir $1
STAR-2.7.10b/source/STAR --runThreadN 31 --genomeDir /work/fastq/STAR38 --readFilesIn $1'_R1_001.fastq.
gz' $1'_R2_001.fastq.gz' --readFilesCommand zcat --outSAMtype BAM SortedByCoordinate --quantMode GeneCounts
--outFileNamePrefix $1'/'$1
```

file: runstar.sh

After testing, alignment of the trimmed RNAseq FASTQ files was then performed for all in Ubuntu (Listing 9) virtual desktop interface with adequate time allotted.

Listing 9) Looping through all sample IDs for the script

2 http://dnatech.genomecenter.ucdavis.edu/faqs/should-i-remove-pcr-duplicates-from-my-rna-seq-data and http://www.biostars.org/p/55648

```
# On 32 vCPU machine with 48 hours reserved
for f in *_trim; do runstar.sh $f; done

# Wait an hour after completion and then shut down the virtual machine hard
sleep 3200 && for i in {1..10}*; do sudo pkill -9 '$i*'; done
```

After alignment, a final quality check was performed for the generated BAMs with FASTQC (Listing 10).

Listing 10) Using “multi-threaded“ FASTQC on a 64 vCPU

```
sudo apt-get update && sudo apt-get install fastqc
fastqc *_trim/*.bam -t 63
```

Documentation for FASTQC: www.bioinformatics.babraham.ac.uk/projects/fastqc

Trimming resulted in an almost complete elimination of the bias (Listing 11) with a read length of 89 bases and 49% GC content (Listing 12).

Listing 11) Representative result of 5’ and trimming

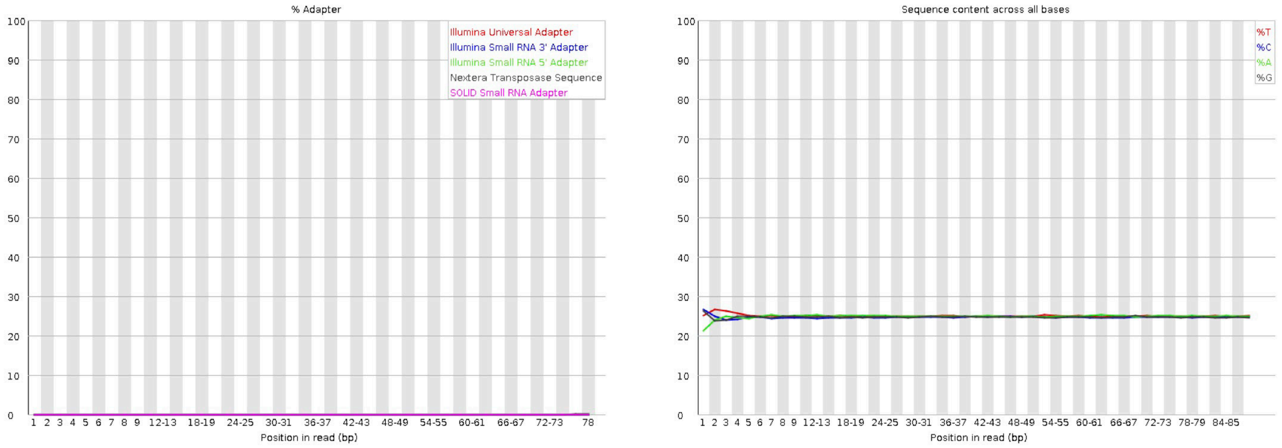

Listing 12) Representative read length, GC content and number of reads (found in FASTQC output)

Measure	Value
Filename	23_0017_S3_trimAligned.sortedByCoord.out.bam
File type	Conventional base calls
Encoding	Sanger / Illumina 1.9
Total Sequences	119247152
Sequences flagged as poor quality	0
Sequence length	89
%GC	49

Because the STAR2-generated BAM files are not directly compatible with HaplotypeCaller (HTC), the GATK command SplitNCigarReads was implemented (Listing 13). As described by the GATK team, this function “*Splits reads that contain Ns in their cigar string (e.g. spanning splicing events in RNAseq data)*”.

Listing 13) Making the STAR2 alignments compatible with GATK

```
#!/bin/bash

samtools index $1'/'$1'Aligned.sortedByCoord.out.bam'

gatk-4.3.0.0/gatk SplitNCigarReads -R resources_broad_hg38_v0_Homo_sapiens_assembly38.fasta -I $1'/'$1'Alig-
ned.sortedByCoord.out.bam' -O $1'/'$1'.bam' -L hg38.bed &
```

file: runBAMFormat.sh

The intervals file was incorporated into the workflow to narrow down the analyses to autosomes and sex chromosomes (Listing 14). This was also done to make the GATK index/reference directly compatible with the STAR2 reference.

Listing 14) Generating a compatible interval file for autosomes, X, and Y

```
awk '/^chr[0-9,X,Y]*\t/ {printf("%s\t0\t%s\n",$1,$2);}' resources_broad_hg38_v0_Homo_sapiens_assembly38.fa-
sta.fai > hg38.bed
```

Total reads per file using were retrieved for plotting fast using samtools view (Listing 15) (16 vCPU machine).

Listing 15) Multithreaded outputting number of reads for plotting

```
sudo apt-get update
sudo apt-get install samtools -y

# Counts for plotting
for f in *_trim/*.recal.bam; do samtools view $f -c -@15; done

# Sample IDs for plotting
for folder in *_trim/; do echo ${folder/_trim\//}; done
```

The output was sorted and imported into GraphPad Prism for distribution plotting of total number of effectively aligned reads (Listing 16).

Listing 16) Read distribution plots generated from samtools view output

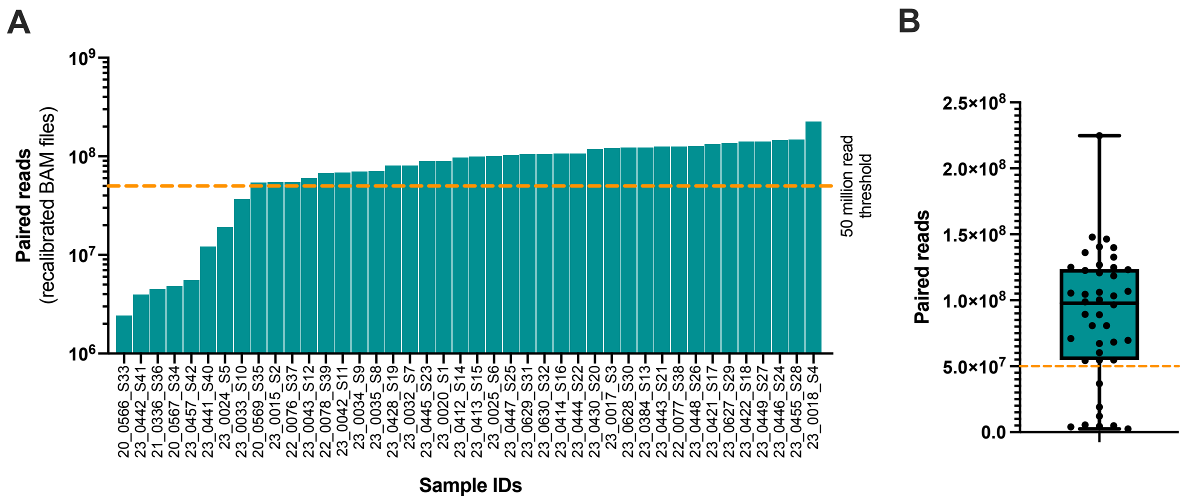

Read statistics were retrieved with samtools stats and summarized using Mathematica (Listing 17).

Listing 17) Getting general read statistics

```
# Assuming that samtools is installed

# Get read statistics for Mathematica and save the first 46 lines for each BAM-file
for f in *_trim/*.recal.bam; do samtools stats $f -@8|(head -n 46)>>stats.txt; done

# Wolfram script for retrieving the different quality parameters, e.g. for sample 2,
# and plotting the median mean quality (field 39):

# sample=3;
# linesperrecord=46;
# display from =10;
# line=(sample*linesperrecord-(linesperrecord-field));
# outp=Flatten[#]&/@Transpose[{Range[field,linesperrecord,1],d[[line;;sample*linesperrecord]]}];
# Part[outp,All,{1,3,4}]//TableForm

# Median[#[[39]][[3]] & /@ Partition[d, 46]]
# BoxWhiskerChart[#[[39]][[3]] & /@ Partition[d, 46]]

# Example output...
#Field      Parameter                              Value
#...10      sequences:                             31573832
#...14      reads mapped:                          31573832
#15         reads mapped and paired:               31573832
#...17      reads properly paired:                 31573832
#...22      non-primary alignments:                6108246
#23         supplementary alignments:              16592194
#24         total length:                          2810071048
#25         total first fragment length:           1405035524
#26         total last fragment length:            1405035524
#27         bases mapped:                          2810071048
#28         bases mapped (cigar):                  2797373393
#...33      average length:                        89
#...39      average quality:                       31.7
#40         insert size average:                   1227.8
#41         insert size standard deviation:        1759.9
#...46      percentage of properly paired reads (%):   100.
```

Using this approach, the general statistics was: (a) median average quality of 26.3, (b) median sequences of 63 millions per sample, 2.4 billion in total, (c) 210 Gb in total using on lane compared to the 1000–1250 Gb output of S2 flow cell, (d) median average insert size (fragment length) of 1056 bp. These figures provide a

estimated mean coverage of 85–93x if using a transcriptome size of 30–33 Mb (C = LN / G), which is, however, very crude or of limited use for RNA-sequencing because expression varies.

For quality score recalibration and variant calling, GATK requires a valid read group tag, RG. Consequently, before base quality score recalibration was performed AddOrReplaceReadGroups was used to add this information (Listing 18).

Listing 18) Adding read group information and performing base quality score recalibration

```
#!/bin/bash
set -x

fn=$1

# Remember to install samtools before running
# sudo apt update
# sudo apt -y install samtools

gatk-4.3.0.0/gatk AddOrReplaceReadGroups -I $fn'_trim/'$fn'_trim.bam' -O $fn'_trim/'$fn'.bam' \
-RGID $fn -RGLB lib1 -RGPL ILLUMINA -RGPU unit1 -RGSM $fn

samtools index $fn'_trim/'$fn'.bam' -@ 4

gatk-4.3.0.0/gatk BaseRecalibrator \
-I $fn'_trim/'$fn'.bam' \
-R /work/hg38/resources_broad_hg38_v0_Homo_sapiens_assembly38.fasta \
--known-sites /work/hg38/resources_broad_hg38_v0_1000G_phase1.snps.high_confidence.hg38.vcf.gz \
--known-sites /work/hg38/resources_broad_hg38_v0_Homo_sapiens_assembly38.dbsnp138.vcf \
--known-sites /work/hg38/resources_broad_hg38_v0_Homo_sapiens_assembly38.known_indels.vcf.gz \
--known-sites /work/hg38/resources_broad_hg38_v0_Mills_and_1000G_gold_standard.indels.hg38.vcf.gz \
-O $fn'_trim/'$fn'.recal.table'

gatk-4.3.0.0/gatk ApplyBQSR \
-I $fn'_trim/'$fn'.bam' \
-R /work/hg38/resources_broad_hg38_v0_Homo_sapiens_assembly38.fasta \
--bqsr-recal-file $fn'_trim/'$fn'.recal.table' \
-O $fn'_trim/'$fn'.recal.bam'
```

file: recalibrate.sh

Variant calling was performed on 4 vCPU machine (duration approx. 48 hours) on intervals identical with the Twist Bioscience exomes. This was done because only variants in these regions are relevant for subsequent pairing with deep sequenced coding CLL genomes (Listing 19). Following indexing soft variant filtration was performed to flag variants with low depth of coverage and low quality. Thus, no variants were removed, only flagged.

Listing 19) Variant calling and flagging

```
#!/bin/bash

fn=$1

gatk-4.3.0.0/gatk --java-options "-Xmx20g" HaplotypeCaller --native-pair-hmm-threads 4 -R /work/hg38/resour-
ces_broad_hg38_v0_Homo_sapiens_assembly38.fasta -L /work/hg38/Twist_Exome_Core_Covered_Targets_hg38.bed -I
$fn'_trim/'$fn'.recal.bam' -O $fn'_trim/'$fn'.vcf'

gatk-4.3.0.0/gatk IndexFeatureFile -I $fn'_trim/'$fn'.vcf'

gatk-4.3.0.0/gatk VariantFiltration \
-R /work/hg38/resources_broad_hg38_v0_Homo_sapiens_assembly38.fasta \
-V $fn'_trim/'$fn'.vcf' \
-O $fn'_trim/'$fn'.filtered.dp100.vcf' \
--filter "QUAL < 30.0" --filter-name "QUAL30" \
--filter-name "Low_depth" \
--filter-expression "DP < 30"
```

Retrival of the number of variants for each sample (Listing 20) showed that 11–12 samples contained a lower amount than the rest of the group (Listing 21). In comparison, a typical WES contains approximately 20.000 variants.

Listing 20) Getting the number of raw and filtered variants for plotting

```
# Print approx. number of variants
for f in *trim/*.dp100.vcf; do awk 'NF > 2' $f|wc -l; done

# Print approx. number of variants with PASS flag
for f in *trim/*.dp100.vcf; do awk 'NF > 2' $f | grep PASS | wc -l; done

# Print Sample IDs
for folder in *_trim/; do echo ${folder/_trim\//}; done
```

Listing 21) Variants for each RNA-sequenced sample

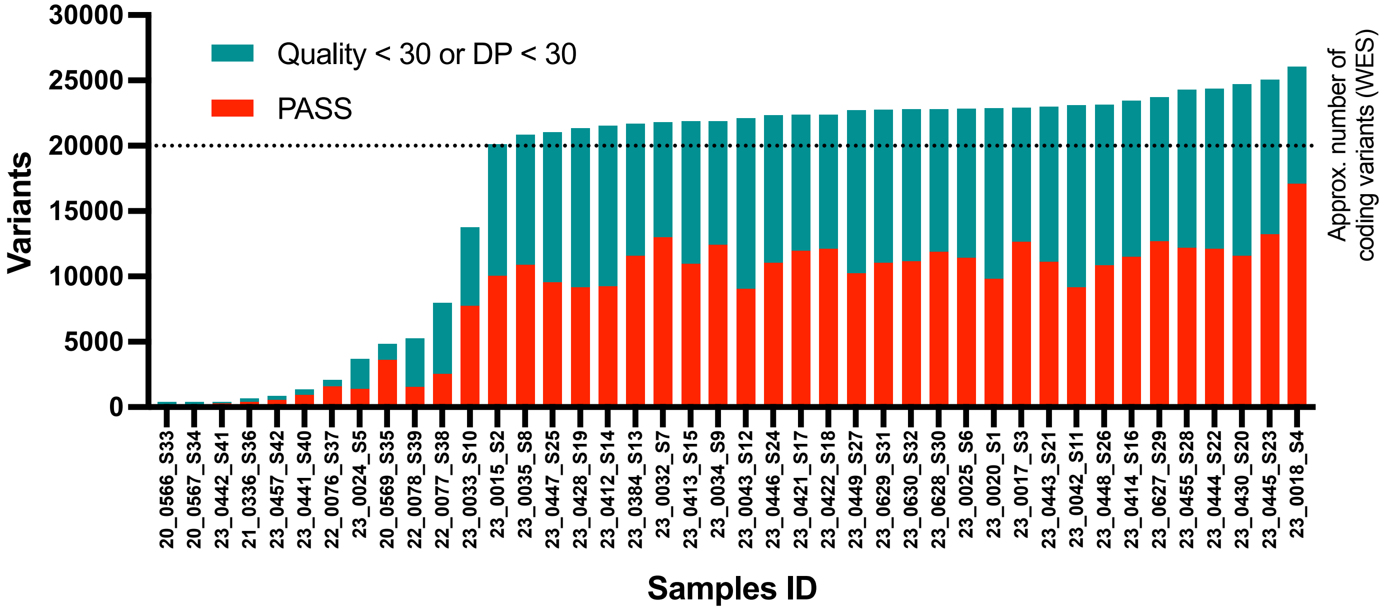

Dimensional reduction using the features sample type, patient ID, RNA volume, RNA concentration (ng/μl), RIN value, eluate volume, RNA amount (ng), treatment, total reads, and number of variants (Listing 22) demonstrated a grouping of the samples into three clusters: One cluster of lower quality diagnostic blood samples (red), and two other clusters of higher quality (Listing 23).

Listing 22) Wolfram script of dimensional reduction and UMAP plotting

```
SetDirectory[NotebookDirectory[]];

(* Importing sample info, and number of reads and variants per sample *)
samples=Select[Import[”RNAsamples.xlsx”][[1]],#[[1]]!=””&][[2;;-1]];
reads=Flatten[{a=StringSplit[#[[1]],”_”][[1]]<>”_”<>StringSplit[#[[1]],”_”][[2]],#}]&/@Select[Import[”RNA-
reads.xlsx”][[1]],#[[1]]!=””&][[2;;-1]];
variants=Flatten[{a=StringSplit[#[[1]],”_”][[1]]<>”_”<>StringSplit[#[[1]],”_”][[2]],#}]&/@Select[Import[”R-
NAvariants.xlsx”][[1]],#[[1]]!=””&][[2;;-1]];

(* Preparing features for ploting *)
features=Flatten[#]&/@GatherBy[Join[samples,reads,variants],First];
features=Flatten[{#[[1]],#[[2]],#[[3]],#[[4]],#[[6;;10]],#[[13]],#[[16;;-1]]}]&/@features;
features//TableForm

(* Performing dimensional reduction and plotting of UMAP *)
reduced = DimensionReduce[Part[features,All,2;;-1], 2, Method->”UMAP”];
ListPlot[MapThread[Labeled[#1, #2]&, {reduced, Part[features,All,1]}],ImageSize->1200]
```

source file: comparesamples.nb (Wolfram Mathematica v. 13.3.1)

The samples of low sequencing quality (PBMC) is thus directly a result of low concentration, RNA amount, and RNA integrity (shown in red) (**Listing 24**). The RIN value was not retrieved for three samples, thus marked as 0 RIN (*) in the table (**Listing 24**).

Listing 23) UMAP of samples features

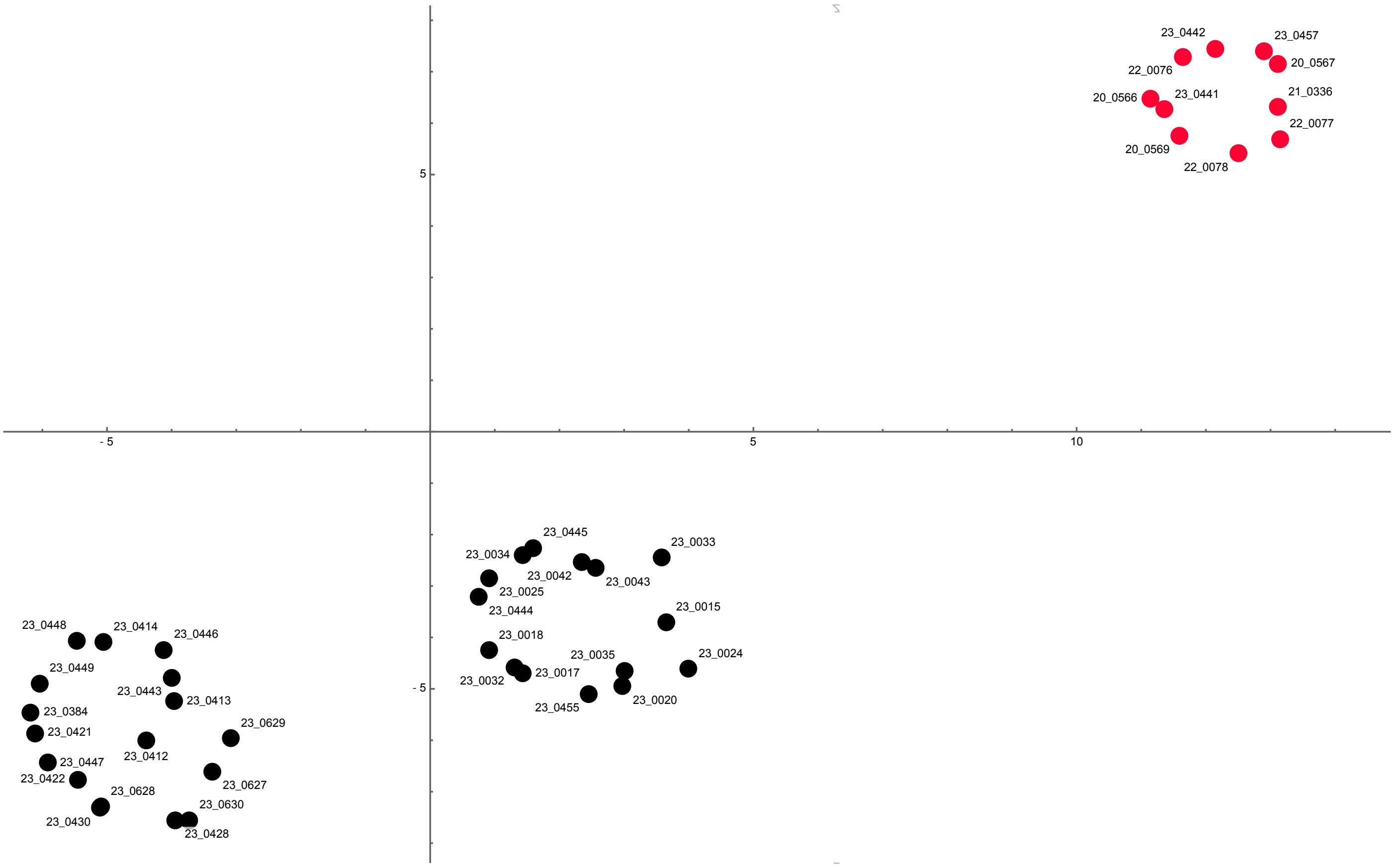

Listing 24) RNA amount and integrity distributions

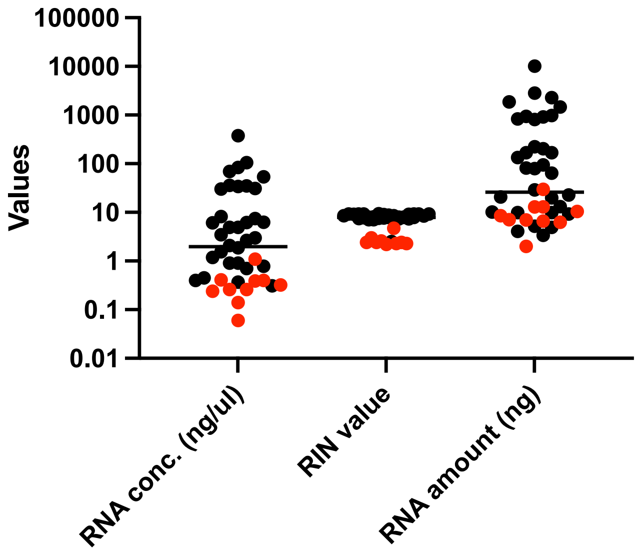

	RNA conc. (ng/µll)	RIN value	RNA amount (ng)
Number of values	42	42	42
Minimum	0.06000	0.000*	2.000
25% Percentile	0.4000	2.575	9.025
Median	1.980	7.850	26.00
75% Percentile	13.67	8.850	368.9
Maximum	377.0	9.400	10179
Range	376.9	9.400	10177
95% CI of median			
Actual confidence level	95.64%	95.64%	95.64%
Lower confidence limit	0.7800	7.100	10.50
Upper confidence limit	6.120	8.100	133.7
Mean	21.99	6.395	584.9
Std. Deviation	61.18	3.088	1655
Std. Error of Mean	9.440	0.4766	255.3

Veyhe et al. 2024. Supplement. Part IV

10X SINGLE-CELL SEQUENCING OF CLL PATIENTS

**Number of samples sequenced**	12 (4 patient samples from sorted B cells, 3 follow-up samples each).<br>Samples were isolated/sorted and fixed (CG000478)<br>Library preparations were performed using Chromium Fixed RNA Profiling Reagent Kits/ (CG000527/Rev D). Dept. of Genetics sequencing project no. "G147-2023 CLL projekt"

Sequencing details
Sequenced on NovasSeq 6000, S4 flow cell, Lane 2. Flow-cell layout as shown.

No.	Patient ID	Sample	Human WTA Probes	POOL	Adaptor
1	664	0026	BC001	Pool 1	1E
2	705	0028	BC002		
3	623	0033	BC003		
4	623	0034	BC004		
5	620	0020P	BC001	Pool 2	1F
6	620	0021P	BC002		
7	664	0022P	BC003		
8	664	0036	BC004		
9	705	0027	BC001	Pool 3	1G
10	620	0029	BC002		
11	623	0035	BC003		
12	705	0037	BC004		

Compiled by Marcus Høy Hansen, marcus.hoy.hansen@rsyd.dk

A total of 12 sorted B-cell samples from four patients undergoing ibrutinib treatment were subjected to single-cell RNA sequencing at three different time points: 13–14 months, 21–24 months, and 35–38 months. The cell viability after B cell isolation measured by flow cytometry was a median of 95.25% (79.6–99.1%).

The single-cell profiling was based on the 10x Chromium Fixed RNA for multiplexed samples (**Listing 1**). The setup involved cells derived from 4 patients with 3 follow-up time points during ibrutinib treatment (**Listing 2**). Prior to fixation the cell populations had been extensively characterized (see appendix table). A median of 3,405,000 cells (range 1,840,000 to 10,700,000 cells) were fixed using the Chromium Next GEM Single Cell Fixed RNA Sample Preparation Kit (10x Genomics, Pleasanton, CA, USA) following the manufacturer's protocol (CG000478) for long-term storage at −80°C. Prior to sequencing, the fixed cells were thawed according to the manufacturer's protocol.

Listing 1) Workflow Overview, Chromium Fixed RNA Profiling for Multiplexed Samples

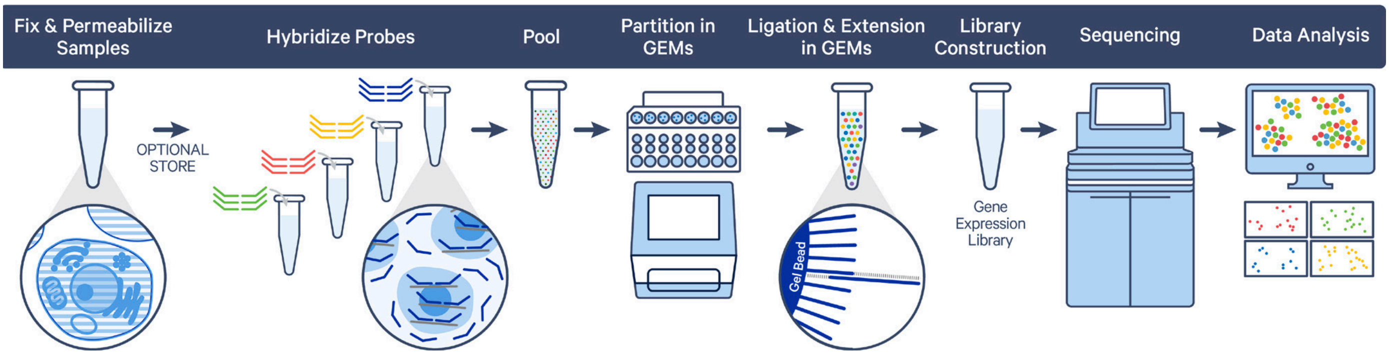

Source: Protocol Planner, CG000528, Rev A, 10x, accessed May 24

Listing 2) Follow-up sample timepoints

Patient ID	Sample ID	Months	Exome sample	Sample year	RNA sequencing
**664**	0026	24.3	0523	2021	0447
**705**	0028	35.1	0603	2022	0020
**623**	0033	13,5	0413	2020	0384
**623**	0034	22,6	0319	2021	0421
**620**	0020P	8.5, 11.0, 13,0, all 1 day old	0503, 0012, 0129	2020, 2021, 2021	0414
**620**	0021P	21.3, 24.2	0674, 0094	2021, 2022	0448
**664**	0022P	31.9, 35.6, 39.7	0210, 0474, 0025, 0837	2022, 2022, 2022	-
**664**	0036	14.5	0543	2020	0422
**705**	0027	23.3	0582	2021	0443
**620**	0029	42.4	0535	2023	-
**623**	0035	38.1	0625	2022	0446
**705**	0037	17.5	0175	2021	-

The sequencing libraries were prepared in three pools (Department of Genetics, Odense University Hospital), with the listed parameters (**Listing 3**) collected prior loading.

Listing 3) Sequencing laboratory library parameters

**Library pool**	**1**	**2**	**3**
Concentration (ng/µl)	10.9	14.2	22.8
Fragment length	257	254	251
Amplicon pool conc. (nM)	64.59	85.14	138.34
	Diluted to 4 nM		
Pool (µl)	3.10	2.35	1.45
H_2O (µl)	46.90	47.65	48.55
Final (µl)	50.00	50.00	50.00

Bioinformatics

The bioinformatics pipeline adopted the 10X cellranger multi pipeline (cellranger-7.2.0). Sequence processing was performed on the UCloud interactive HPC system, managed by the eScience Center at the University of Southern Denmark (Ubuntu Xfce virtual desktop environment 22.04). Downstream analysis was performed in RStudio (Version 2023.09.1+494) running R version 4.3.1 with Seurat (5.0.3), SeuratObject (5.0.1), LoupeR (1.0.2), cluster (2.1.6) and ggplot2 (3.5.0), future (1.33.2) packages. FASTQ files were processed for each of the three library pools (Listing 2), each run with a separat configuration file for the used barcodes (See (Listing 4), (Listing 5) and (Listing 6)).

Listing 4) Running cellranger multi for all 3 pools

```
#!/bin/bash

cellranger-7.2.0/cellranger multi --id cll_10xpool1 --csv cll_10xpool1.csv --localvmem 200 --localmem 200
--localcores 16

cellranger-7.2.0/cellranger multi --id cll_10xpool2 --csv cll_10xpool2.csv --localvmem 200 --localmem 200
--localcores 16

cellranger-7.2.0/cellranger multi --id cll_10xpool3 --csv cll_10xpool3.csv --localvmem 200 --localmem 200
--localcores 16
```

Listing 5) Configuration file for pool 1 (cll_10xpool1.csv)

```
[gene-expression]
ref,/work/CLL_10X24/refdata-gex-GRCh38-2020-A
probe-set,/work/CLL_10X24/Chromium_Human_Transcriptome_Probe_Set_v1.0_GRCh38-2020-A.csv
no-bam,true

[libraries]
fastq_id,fastqs,lanes,physical_library_id,feature_types,subsample_rate
S1,/work/CLL_10X24/H55TKDSXC/pool1,any,sample,Gene Expression,

[samples]
sample_id,probe_barcode_ids,description,
"pt664-0026-BC001",BC001,pt664-0026-BC001
"pt705-0028-BC002",BC002,pt705-0028-BC002
"pt623-0033-BC003",BC003,pt623-0033-BC003
"pt623-0034-BC004",BC004,pt623-0034-BC004
```

Listing 6) Configuration file for pool 2 (cll_10xpool2.csv)

```
[gene-expression]
ref,/work/CLL_10X24/refdata-gex-GRCh38-2020-A
probe-set,/work/CLL_10X24/Chromium_Human_Transcriptome_Probe_Set_v1.0_GRCh38-2020-A.csv
no-bam,true

[libraries]
fastq_id,fastqs,lanes,physical_library_id,feature_types,subsample_rate
S2,/work/CLL_10X24/H55TKDSXC/pool2,any,sample,Gene Expression,

[samples]
sample_id,probe_barcode_ids,description,
"pt620-0020P-BC001",BC001,pt620-0020P-BC001
"pt620-0021P-BC002",BC002,pt620-0021P-BC002
"pt664-0022P-BC003",BC003,pt664-0022P-BC003
"pt664-0036-BC004",BC004,pt664-0036-BC004
```

Listing 7) Configuration file for pool 3 (cll_10xpool3.csv)

```
[gene-expression]
ref,/work/CLL_10X24/refdata-gex-GRCh38-2020-A
probe-set,/work/CLL_10X24/Chromium_Human_Transcriptome_Probe_Set_v1.0_GRCh38-2020-A.csv
no-bam,true

[libraries]
fastq_id,fastqs,lanes,physical_library_id,feature_types,subsample_rate
S3,/work/CLL_10X24/H55TKDSXC/pool3,any,sample,Gene Expression,

[samples]
sample_id,probe_barcode_ids,description,
705-0027-BC001,BC001,705-0027-BC001
620-0029-BC002,BC002,620-0029-BC002
623-0035-BC003,BC003,623-0035-BC003
705-0037-BC004,BC004,705-0037-BC004
```

The total number of reads acquired for analyses was ~5.8 billion with a high fraction of cell barcode/UMI bases with Q-score ≥30 (98.6–98.8%) (Listing 8). The median number of cells for each barcode was 14,541 (4,146–21,417) with an average median number of reads per cell at 283,193 (9,743–45,270) (Listing 9)-11).

Listing 8) Quality metrics for each sequencing pool

Pool	1	2	3
**Adapter**	**1E**	**1F**	**1G**
Number of reads	1929949211	1655209725	2250355970
Number of short reads skipped	0	0	0
Q30 GEM barcodes	98.8%	98.8%	98.6%
Q30 RNA read	98.5%	98.5%	98.3%
Q30 UMI	98.8%	98.8%	98.7%
Q30 barcodes	98.3%	98.4%	98.1%
Q30 probe barcodes	97.2%	97.7%	97.1%
Confidently mapped reads in cells	96.83%	96.66%	97.20%
Estimated number of cells	53742	4614	65853
Fraction of initial cell barcodes passing high occupancy GEM filtering	100.00%	99.84%	100.00%
Mean reads per cell	35911	35874	34172
Number of reads	1929949211	1655209725	2250355970
Number of reads in the library	1929949211	1655209725	2250355970
Reads confidently mapped to filtered probe set	92.90%	92.64%	93.11%
Reads confidently mapped to probe set	95.19%	94.75%	95.84%
Reads half-mapped to probe set	0.88%	1.02%	0.75%
Reads mapped to probe set	96.89%	97.00%	97.28%
Reads split-mapped to probe set	0.81%	1.23%	0.69%
Sequencing saturation	88.68%	84.73%	83.33%
Valid GEM barcodes	98.96%	98.97%	98.98%
Valid UMIs	100.00%	100.00%	100.00%
Valid barcodes	96.01%	96.08%	96.49%
Valid probe barcodes	96.92%	97.00%	97.40%
Cells per probe barcode	11925 (22.19%)	5581 (12.10%)	21417 (32.52%)

Sample ID	pt664-0026-BC001	pt620-0020P-BC001	705-0027-BC001
UMIs per probe barcode	74436407 (36.56%)	10717200 (4.58%)	110727846 (31.47%)
Cells per probe barcode	8140 (15.15%)	4146 (8.99%)	10456 (15.88%)
Sample ID	pt705-0028-BC002	pt620-0021P-BC002	620-0029-BC002
UMIs per probe barcode	34273194 (16.83%)	21316392 (9.10%)	56020011 (15.92%)
Cells per probe barcode	13296 (24.74%)	18995 (41.17%)	15786 (23.97%)
Sample ID	pt623-0033-BC003	pt664-0022P-BC003	623-0035-BC003
UMIs per probe barcode	40197797 (19.74%)	147282001 (62.88%)	54914525 (15.61%)
Cells per probe barcode	20381 (37.92%)	17418 (37.75%)	18194 (27.63%)
Sample ID	pt623-0034-BC004	pt664-0036-BC004	705-0037-BC004
UMIs per probe barcode	54415552 (26.73%)	54492460 (23.27%)	129747358 (36.88%)

Listing 9) Pool 1 barcode metrics

Pool 1	Barcode 1 (BC001)	Barcode 2 (BC002)	Barcode 3 (BC003)	Barcode 4 (BC004)
Cells	11925	8140	13296	20381
Confidently mapped reads in cells	97.41%	95.24%	97.33%	96.97%
Estimated UMIs from genomic DNA	-	-	-	-
Estimated UMIs from genomic DNA per unspliced probe	-	-	-	-
Median UMI counts per cell	4820	3119	2608	2142
Median genes per cell	2557	1828	1578	1342
Median reads per cell	45270	29294	24375	19864
Number of reads from cells called from this sample	655251828	300851315	356042513	477628322
Reads confidently mapped to filtered probe set	95.02%	94.48%	95.25%	95.18%
Reads confidently mapped to probe set	97.52%	96.53%	97.59%	97.44%
Reads half-mapped to probe set	0.20%	0.23%	0.19%	0.20%
Reads mapped to probe set	98.24%	98.10%	98.26%	98.16%
Reads split-mapped to probe set	0.53%	1.34%	0.48%	0.52%
Total genes detected	15144	15212	13949	14895
Cells detected in other samples	41817 (77.81%)	45602 (84.85%)	40446 (75.26%)	33361 (62.08%)
Cells detected in this sample	11925 (22.19%)	8140 (15.15%)	13296 (24.74%)	20381 (37.92%)
Number of reads	1929949211	1929949211	1929949211	1929949211
Number of short reads skipped	0	0	0	0

Listing 10) Pool 2 barcode metrics

Pool 2	Barcode 1 (BC001)	Barcode 2 (BC002)	Barcode 3 (BC003)	Barcode 4 (BC004)
Cells	5581	4146	18995	17418
Confidently mapped reads in cells	89.18%	96.31%	97.24%	97.26%
Estimated UMIs from genomic DNA	-	-	-	-
Estimated UMIs from genomic DNA per unspliced probe	-	-	-	-
Median UMI counts per cell	1387	4234	4406	2451
Median genes per cell	960	2283	2356	1565
Median reads per cell	9743	29202	30910	16901
Number of reads from cells called from this sample	66822295	138112418	970880078	351655178
Reads confidently mapped to filtered probe set	91.93%	95.26%	95.19%	95.19%
Reads confidently mapped to probe set	93.67%	97.64%	97.24%	97.66%
Reads half-mapped to probe set	0.30%	0.19%	0.20%	0.20%
Reads mapped to probe set	97.78%	98.24%	98.20%	98.10%
Reads split-mapped to probe set	3.81%	0.41%	0.76%	0.24%
Total genes detected	14308	14722	16134	14108
Cells detected in other samples	40559 (87.90%)	41994 (91.01%)	27145 (58.83%)	28722 (62.25%)
Cells detected in this sample	5581 (12.10%)	4146 (8.99%)	18995 (41.17%)	17418 (37.75%)

Listing 11) Pool 3 barcode metrics

Metric Name	Barcode 1 (BC001)	Barcode 2 (BC002)	Barcode 3 (BC003)	Barcode 4 (BC004)
Cells	21417	10456	15786	18194
Confidently mapped reads in cells	97.54%	96.86%	96.72%	97.46%
Estimated UMIs from genomic DNA	-	-	-	-
Estimated UMIs from genomic DNA per unspliced probe	-	-	-	-
Median UMI counts per cell	4193	4643	2980	5461
Median genes per cell	2237	2394	1796	2671
Median reads per cell	26668	29470	18946	34921
Number of reads from cells called from this sample	664492275	335870262	328769447	777347443
Reads confidently mapped to filtered probe set	95.08%	94.95%	95.25%	94.04%
Reads confidently mapped to probe set	97.64%	97.32%	97.33%	97.43%
Reads half-mapped to probe set	0.19%	0.20%	0.20%	0.20%

**Reads mapped to probe set**	98.23%	98.29%	98.21%	98.19%
**Reads split-mapped to probe set**	0.41%	0.77%	0.68%	0.56%
**Total genes detected**	15739	15785	14758	16067
**Cells detected in other samples**	44436 (67.48%)	55397 (84.12%)	50067 (76.03%)	47659 (72.37%)
**Cells detected in this sample**	21417 (32.52%)	10456 (15.88%)	15786 (23.97%)	18194 (27.63%)

Final analyses were performed in R and Seurat (Listing 12) (Listing 13). Figures was further prepared and set in GraphPad Prism (10.1) and Adobe Illustrator (Adobe Creative Cloud 6.1.0).

Listing 12) R code

```
rm(list = ls())
library(Seurat)
library(ggplot2)
options(future.globals.maxSize = 2000 * 1024^2)

setwd("HPF/10X/")
library(future)
plan("multisession", workers = 8) # Adjust the number of workers based on your machine

# **** DEFINED FUNCTIONS ****

Load10x <- function(fi,samname) {
  s <- Read10X_h5(paste(fi,"_sample_filtered_feature_bc_matrix.h5",sep = ""),
use.names = TRUE, unique.features = TRUE)
  sample <- CreateSeuratObject(counts = s, project = "CLL24", min.cells = 10, min.features = 500)
  sample$orig.ident <- samname
  sample <-subset(x = sample, downsample = 1000)
  return(sample)
}

MergeNormFilter <- function(s1,s2,s3) {
  mergedSamples <- merge(s1, y = c(s2,s3), project = "CLLMerged")
  mergedSamples<- NormalizeData(mergedSamples)
  mergedSamples<- FindVariableFeatures(mergedSamples, selection.method = "vst", nfeatures = 2000)
  mergedSamples[["percent.mt"]] <- PercentageFeatureSet(mergedSamples, pattern = "^MT-")
  filteredSeuratObject <-
subset(mergedSamples, subset = nFeature_RNA > 200 & nFeature_RNA < 5000 & percent.mt < 5)
  all.genes <- rownames(filteredSeuratObject)
  filteredSeuratObject <- ScaleData(filteredSeuratObject, features = all.genes)
  filteredSeuratObject <-
RunPCA(filteredSeuratObject, features = VariableFeatures(object = filteredSeuratObject))
  filteredSeuratObject <- FindNeighbors(filteredSeuratObject, dims = 1:10)
  filteredSeuratObject <- RunUMAP(filteredSeuratObject, dims = 1:10)
  return(filteredSeuratObject)
}

SelectBcells <- function(Sobj,clu=NULL, res=0.3,plotmode=3) {
  sampleO <- FindClusters(Sobj, resolution = res)

  if(plotmode==1) {
    r<-DimPlot(sampleO, reduction = "umap", label = TRUE)
  } else if(plotmode==2) {
    r<-DimPlot(subset(sampleO, idents = clu, invert = TRUE), reduction = "umap", label = TRUE)
  } else {
    r<-subset(sampleO, idents = clu, invert = TRUE)
  }
  return(r)
}

getX <- function(g) {

  a<-FetchData(EarlyR, vars = g)[,1];
  b<-FetchData(LateR, vars = g)[,1];
```

```
  rlist<-c(
    c("Early mean",mean(a)),
    c("Late mean",mean(b)),
    c("Early median",median(a)),
    c("Late median",median(b)),

    c("Early mean > 0",mean(a[a>0])),
    c("Late mean > 0",mean(b[b>0])),
    c("Early median > 0",median(a[a>0])),
    c("Late median > 0",median(b[b>0])),
    c("Relative length ratio > 0: ",length(b[b>0])/length(a[a>0])),
    c("Relative length ratio",length(b)/length(a))
  )
  print(rlist)
}

# **********************************************
patient<-"620"
# **********************************************

s1<-Load10x("pt620-0020P-BC001", "sample 620-1")
s2<-Load10x("pt620-0021P-BC002", "sample 620-2")
s3<-Load10x("620-0029-BC002", "sample 620-3")

# **********************************************

sampleO <- MergeNormFilter(s1,s2,s3)
VlnPlot(sampleO, features = c("nFeature_RNA", "nCount_RNA", "percent.mt"), ncol = 3,group.by = "orig.ident")


# To test parameters
#plot<-SelectBcells(sampleO,NULL,0.3,1)
#plot<-SelectBcells(sampleO, c(3),0.3,2)

sampleBcells<-SelectBcells(sampleO, c(2,3,5,6),0.3)
DimPlot(sampleBcells, reduction = "umap", group.by = "orig.ident", label = TRUE)


filen<-paste("sampleBcells_",patient,".rds",sep="")
saveRDS(object = sampleBcells,file = filen)


# **********************************************
patient<-"623"
# **********************************************

s1<-Load10x("pt623-0033-BC003", "sample 623-1")
s2<-Load10x("pt623-0034-BC004", "sample 623-2")
s3<-Load10x("623-0035-BC003", "sample 623-3")

# **********************************************

sampleO <- MergeNormFilter(s1,s2,s3)
VlnPlot(sampleO, features = c("nFeature_RNA", "nCount_RNA", "percent.mt"), ncol = 3,group.by = "orig.ident")


sampleBcells<-SelectBcells(sampleO, c(3),0.3)
DimPlot(sampleBcells, reduction = "umap", group.by = "orig.ident", label = TRUE)

filen<-paste("sampleBcells_",patient,".rds",sep="")
saveRDS(object = sampleBcells,file = filen)


# **********************************************
patient<-"664"
# **********************************************

s1<-Load10x("pt664-0036-BC004", "sample 664-1")
s2<-Load10x("pt664-0026-BC001", "sample 664-2")
s3<-Load10x("pt664-0022P-BC003", "sample 664-3")

# **********************************************

sampleO <- MergeNormFilter(s1,s2,s3)
VlnPlot(sampleO, features = c("nFeature_RNA", "nCount_RNA", "percent.mt"), ncol = 3,group.by = "orig.ident")

sampleBcells<-SelectBcells(sampleO, c(5,6),0.5)
```

```
DimPlot(sampleBcells, reduction = "umap", group.by = "orig.ident", label = TRUE)

filen<-paste("sampleBcells_",patient,".rds",sep="")
saveRDS(object = sampleBcells,file = filen)


# *********************************************
patient<-"705"
# *********************************************

s1<-Load10x("705-0037-BC004", "sample 705-1")
s2<-Load10x("705-0027-BC001", "sample 705-2")
s3<-Load10x("pt705-0028-BC002", "sample 705-3")

# *********************************************

sampleO <- MergeNormFilter(s1,s2,s3)
VlnPlot(sampleO, features = c("nFeature_RNA", "nCount_RNA", "percent.mt"), ncol = 3,group.by = "orig.ident")

sampleBcells<-SelectBcells(sampleO, c(3,4,5,6,7),0.3)
DimPlot(sampleBcells, reduction = "umap", group.by = "orig.ident", label = TRUE)

filen<-paste("sampleBcells_",patient,".rds",sep="")
saveRDS(object = sampleBcells,file = filen)

# *********************************************


# ***** LOAD SAMPLES AND MERGE TO ONE OBJECT *****
sampleBcells_620 <- readRDS(file = "sampleBcells_620.rds")
sampleBcells_623 <- readRDS(file = "sampleBcells_623.rds")
sampleBcells_664 <- readRDS(file = "sampleBcells_664.rds")
sampleBcells_705 <- readRDS(file = "sampleBcells_705.rds")

mergedSamples <-
merge(sampleBcells_620, y = c(sampleBcells_623, sampleBcells_664, sampleBcells_705), project = "CLL24")
mergedSamples [["RNA"]] <- JoinLayers(mergedSamples[["RNA"]])
Layers(mergedSamples[["RNA"]])


# ***** SCALE, RUN PCA, FIND NEAREST NEIGHBORS, RUN UMAP, AND SAVE OBJECT FOR FUTURE USE *****
all.genes <- rownames(mergedSamples)
mergedSamples <- ScaleData(mergedSamples, features = all.genes)
mergedSamples<- RunPCA(mergedSamples, features = VariableFeatures(object = mergedSamples))
mergedSamples <- FindNeighbors(mergedSamples, dims = 1:10)
mergedSamples <- RunUMAP(mergedSamples, dims = 1:10)

DimPlot(mergedSamples, reduction = "umap", group.by = "orig.ident", label = TRUE)
saveRDS(object = mergedSamples ,file = "sampleBcells_all.rds")


# **** START FROM HERE IF ALREADY SAVED AS RDS! ****
# **** Define early, intermediate, and late response samples ****
mergedSamples <- readRDS(file = "sampleBcells_all.rds")

Idents(object = mergedSamples) <- "orig.ident"
LateR <- subset(x = mergedSamples, idents = c("sample 620-3","sample 623-3","sample 664-3","sample 705-3"))
Idents(object = LateR) <- "Late"

MediumR <- subset(x = mergedSamples, idents = c("sample 620-2","sample 623-2","sample 664-2","sample 705-
2"))
Idents(object = MediumR) <- "Medium"

EarlyR <- subset(x = mergedSamples, idents = c("sample 620-1","sample 623-1","sample 664-1","sample 705-1"))
Idents(object = EarlyR) <- "Early"

DimPlot(LateR, reduction = "umap", group.by = "orig.ident", label = TRUE)
DimPlot(MediumR, reduction = "umap", group.by = "orig.ident", label = TRUE)
DimPlot(EarlyR, reduction = "umap", group.by = "orig.ident", label = TRUE)


# **** MERGE EARLY AND LATE FOR ANALYSES ****
le<-merge(LateR, y = EarlyR, add.cell.ids = c("late","early"))

le[["RNA"]] <- JoinLayers(le[["RNA"]])

all.genes <- rownames(le)
```

```
le <- ScaleData(le, features = all.genes)
le<- RunPCA(le, features = VariableFeatures(object = le))
le <- FindNeighbors(le, dims = 1:10)
le <- RunUMAP(le, dims = 1:10)

saveRDS(object = le ,file = "EarlyLate_merged1.rds")


# **** START FROM HERE IF ALREADY SAVED COMPLETE SET ****
le <- readRDS(file = "EarlyLate_merged1.rds")

# **** PLOT UMAP ****
DimPlot(le, reduction = "umap")
DimPlot(le, reduction = "umap", group.by = "orig.ident", label = TRUE)

# **** SHOW 100 MOST SIGNIFICANT MARKERS  ****
ins<-100
cluster.markers <- FindMarkers(le, ident.1 = "Late", ident.2 = "Early")
df<-cluster.markers
df <- df[abs(df[,3] - df[,4]) > 0.10, ]
df <- df[order(df[,2],decreasing = TRUE), ]
head(df, n = ins)[,-1]
rownames(head(df, n = ins))
DoHeatmap(le, features = rownames(head(df, n = ins)))+theme(text = element_text(size = 6))

# **** GET GENE NAMES FOR GSEA / GENE ONTOLOGY  ****
cat(rownames(head(df, n = ins)), sep = "\n")


# DEFINE CATEGORIES FROM SIGNIFICANT SET
gene_categories <- list(

  NF_kB_Pathway_Regulators_and_Components = c("NFKBIA", "NFKBID", "NFKB2","NFKBIE","NFKBIZ","TNFAIP3"),
  Stress_Response_and_Protein_Phosphatase_Regulation = c("PPP1R15A", "PPP1R15B","IER5","TP53IN-
P1","BCL2L11"),
  Transcription_Factors_and_DNA_RNA_Binding_Proteins = c("KLF6", "IRF1", "KDM2A", "SRSF7", "TRA2B", "TRA2A",
"HNRNPH3", "ZNF394", "RSRP1", "ZC3H15", "RBM14"),
  Mitochondrial_Genes_Oxidative_Phosphorylation = c("MT-ND1", "MT-ND2", "MT-ND3", "MT-ND4", "MT-ND4L", "MT-
ND5", "MT-CYB", "MT-ATP6"),
  Immune_System_and_Cell_Surface_Markers = c("CD83", "CD69", "CD79B", "SLAMF6", "TAPBP", "TCL1A", "FCRLA",
"FCRL5")

)

# Remove categories with only one member
# Print the filtered list

flattened_gene_names <- unlist(gene_categories, use.names = FALSE)
flattened_gene_names
DoHeatmap(le, features = flattened_gene_names )


# **** EXPORTING FOR PLOTS *****
targetarray<-c("NFKBIA","NFKBIZ","NFKBIE","TNFAIP3","PPP1R15A","IER5", "TP53INP1","BCL2L11")

for(s in targetarray) {
  print(s)
  write.table(FetchData(EarlyR, vars = s),paste("early_",s,".tsv", sep=""),sep = "\t")
  write.table(FetchData(LateR, vars = s),paste("late_",s,".tsv", sep=""),sep = "\t")

}


# **** LIST RELATIVE EXPRESSION FOR ASSESSMENT *****
exttargets<-c(targetarray,c("CD5","CD19","MS4A1","FCER2"))
for(s in exttargets) {
  print(paste("*** ",s,": ****"))
  getX(s)

}


# **** PLOTS FOR INDIVIDUAL SAMPLES *****
RidgePlot(le, features = targetarray, ncol = 3,group.by = "orig.ident")
VlnPlot(le, features =  exttargets, ncol = 4,pt.size = 0,group.by = "orig.ident")
```

```
# **** PLOTS FOR EARLY vs LATE SAMPLES *****
RidgePlot(le, features = targetarray, ncol = 3)
VlnPlot(le, features =  exttargets, ncol = 4,pt.size = 0)

FeaturePlot(EarlyR, features =  targetarray,order=TRUE,pt.size = 1 )
FeaturePlot(LateR, features =  targetarray,order=TRUE,pt.size = 0.1 )
FeaturePlot(mergedSamples, features =  targetarray,order=TRUE,pt.size = 0.05 )

FeaturePlot(object = mergedSamples, features = targetarray, cols = c("#4AEB8E66", "#FF5A1266"), pt.size =
0.01)

# **** GET TOP VARIABLE GENES WITHIN SAMPLES: WARNING NOT USED IN PAPER *****
mergedSamplesB<- FindVariableFeatures(mergedSamples, selection.method = "vst", nfeatures = 2000)
top100 <- head(VariableFeatures(mergedSamplesB), 100)
top100

# **** HOW WELL DOES THE CLUSTERING PERFORM IN SILHOUTETTE SCORE *****
library(cluster)
# Extract cluster assignments
cluster_assignments <- Idents(mergedSamples)

# Extract UMAP embeddings
umap_embeddings <- Embeddings(mergedSamples, "umap")

# Calculate silhouette scores. Note: silhouette function expects raw data, but we typically use it with di-
stance matrices or dissimilarity measures. Here we're simplifying the approach to demonstrate the concept.
silhouette_scores <- silhouette(as.numeric(cluster_assignments), dist(umap_embeddings))

# Average silhouette width for each cluster
avg_sil_width <- summary(silhouette_scores)$avg.width

print(avg_sil_width)
DimPlot(le, reduction = "umap")
DimPlot(le, reduction = "umap", group.by = "orig.ident", label = TRUE)
```

Listing 13) Generating heatmap plot

```
# Original list with all categories
gene_categories <- list(
  Stress_Response_and_Protein_Phosphatase_Regulation = c("PPP1R15A", "PPP1R15B","IER5"),
  NF_kB_Pathway_Regulators_and_Components = c("NFKBIA", "NFKBID", "NFKB2", "NFKBIZ"),
  Transcription_Factors_and_DNA_RNA_Binding_Proteins = c("KLF6", "IRF1", "KDM2A", "SRSF7", "TRA2B", "TRA2A",
"HNRNPH3", "ZNF394", "RSRP1", "ZC3H15", "RBM14"),
  Mitochondrial_Genes_Oxidative_Phosphorylation = c("MT-ND1", "MT-ND2", "MT-ND3", "MT-ND4", "MT-ND4L", "MT-
ND5", "MT-CYB", "MT-ATP6"),
  Immune_System_and_Cell_Surface_Markers = c("CD83", "CD69", "CD79B", "SLAMF6", "TAPBP", "TCL1A", "FCRLA",
"FCRL5")

)

# Remove categories with only one member
filtered_gene_categories <- gene_categories[sapply(gene_categories, length) > 1]

# Print the filtered list

flattened_gene_names <- unlist(filtered_gene_categories, use.names = FALSE)
flattened_gene_names
DoHeatmap(le, features = flattened_gene_names )
length(flattened_gene_names)

stress_response_genes <- c("PPP1R15A", "PPP1R15B", "JMY", "RBM14", "RELT", "CCNL1","MARCKS","IER5", "ST-
K17B", "IRF1", "TP53INP1", "NFKB2","SMAD3","HIPK1","BCL2L11","GADD45B","MS4A1","FCER2","CD19","CD5")
stress_response_genes <- c("PPP1R15A", "PPP1R15B", "JMY", "RELT", "CCNL1","MARCKS","IER5", "STK17B", "IRF1",
"TP53INP1", "NFKB2","SMAD3","HIPK1","BCL2L11","GADD45B")

# Print the list
print(stress_response_genes)
VlnPlot(le, features = stress_response_genes, ncol = 4,pt.size = 0,group.by = "orig.ident")
Mitochondrial_Genes_Oxidative_Phosphorylation <- c("MT-ND1", "MT-ND2", "MT-ND3", "MT-ND4", "MT-ND4L", "MT-
ND5", "MT-CYB", "MT-ATP6")

VlnPlot(le, features = Mitochondrial_Genes_Oxidative_Phosphorylation , ncol = 4,pt.size = 0,group.by =
"orig.ident")
```
